# Evolutionary dynamics with game transitions

**DOI:** 10.1073/pnas.1908936116

**Published:** 2019-11-26

**Authors:** Qi Su, Alex McAvoy, Long Wang, Martin A. Nowak

**Affiliations:** ^a^Center for Systems and Control, College of Engineering, Peking University, Beijing 100871, China;; ^b^Program for Evolutionary Dynamics, Harvard University, Cambridge, MA 02138;; ^c^Department of Organismic and Evolutionary Biology, Harvard University, Cambridge, MA 02138;; ^d^Department of Mathematics, Harvard University, Cambridge, MA 02138

**Keywords:** cooperation, evolutionary game theory, game transitions, structured populations

## Abstract

The environment has a strong influence on a population’s evolutionary dynamics. Driven by both intrinsic and external factors, the environment is subject to continual change in nature. To capture an ever-changing environment, we consider a model of evolutionary dynamics with game transitions, where individuals’ behaviors together with the games that they play in one time step influence the games to be played in the next time step. Within this model, we study the evolution of cooperation in structured populations and find a simple rule: Weak selection favors cooperation over defection if the ratio of the benefit provided by an altruistic behavior, b, to the corresponding cost, c, exceeds k−k′, where k is the average number of neighbors of an individual and k′ captures the effects of the game transitions. Even if cooperation cannot be favored in each individual game, allowing for a transition to a relatively valuable game after mutual cooperation and to a less valuable game after defection can result in a favorable outcome for cooperation. In particular, small variations in different games being played can promote cooperation markedly. Our results suggest that simple game transitions can serve as a mechanism for supporting prosocial behaviors in highly connected populations.

## Significance

Evolving populations are constantly subjected to changing environmental conditions. The environment can determine how the expression of traits affects the individuals possessing them. Just as important, however, is the fact that the expression of traits can also alter the environment. We model this phenomenon by introducing game transitions into classical models of evolutionary dynamics. Interacting individuals receive payoffs from the games that they play, and these games can change based on past actions. We find that game transitions can significantly reduce the critical benefit-to-cost threshold for cooperation to evolve in social dilemmas. This result improves our understanding of when cooperators can thrive in nature, even when classical results predict a high critical threshold.

The prosocial act of bearing a cost to provide another individual with a benefit, which is often referred to as “cooperation” (1), reduces the survival advantage of the donor and fosters that of the recipient. Understanding how such a trait can be maintained in a competitive world has long been a focal issue in evolutionary biology and ecology (2). The spatial distribution of a population makes an individual more likely to interact with neighbors than with those who are more distant. Population structures can affect the evolution of cooperation (3–9). In “viscous” populations, one’s offspring often stay close to their places of birth. Relatives thus interact more often than 2 random individuals. Compared with the well-mixed setting, population “viscosity” is known to promote cooperation (10) [although when the population density is fixed, local competition can cancel the cooperation-promoting effect of viscosity (11, 12)]. Past decades have seen an intensive investigation of the evolution of cooperation in graph-structured populations (6–9). One of the best-known findings is that weak selection favors cooperation if the ratio of the benefit provided by an altruistic act, b, to the cost of expressing such an altruistic trait, c, exceeds the average number of neighbors, k (i.e., b/c>k) (6, 13). This simple rule strongly supports the proposition that population structure is one of the major mechanisms responsible for the evolution of cooperation (2).

However, many realistic systems are highly connected, with each individual having many neighbors on average. For example, in a contact network consisting of students from a French high school, each student has 36 neighbors on average, meaning k=36 (14). In such cases, the threshold for establishing cooperation, based on the rule “b/c>k,” is quite high: the benefit from an altruistic act must be at least 36 times greater than its cost. Somewhat large mean degrees have also been observed in collegiate Facebook networks, with well-known examples ranging from 39 neighbors to well over 100 (15–17). Such networks can (and do) involve the expression of social behaviors much more complex than those captured by the simple model of cooperation described previously. However, even for such a simple model, it is not understood if and when the threshold for the evolution of cooperation can be reduced to something less than the mean number of neighbors. Here, we consider a natural way in which this threshold can be relaxed using “game transitions.”

In evolutionary game theory, an individual’s reproductive success is determined by games played within the population. Many prior studies have relied on an assumption that the environment in which individuals evolve is time invariant, meaning that the individuals play a single fixed game. However, this assumption is not always realistic and can represent an oversimplification of reality (18), as many experimental studies have shown that the environment that individuals face changes over time (and often) (19–22). As a simple example, overgrazing typically leads to the degradation of the common pasture land, leaving herders with fewer resources to utilize in subsequent seasons. By constraining the number of livestock within a reasonable range, herders can achieve a more sustainable use of pasture land (23). In this kind of population, individuals’ actions influence the state of environment, which in turn, impacts the actions taken by its members. Apart from endogenous factors, like individuals’ actions, exogenous factors, like seasonal climate fluctuations and soil conditions, can also modify the environment experienced by the individuals. Examples are not limited to human-related activities but also appear in various microbial systems, including bacteria and viruses (21, 22).

In this study, we use graphs to model a population’s spatial structure, where nodes represent individuals and edges describe their interactions. We propose a model of evolutionary dynamics with game transitions: individuals sharing an edge interact (“play a game”) in each time step, and their strategic actions together with the game played determine the game to be played in the next time step. We find that game transitions can lower the threshold for establishing cooperation by k′, which means that the condition for cooperation to evolve is b/c>k−k′, where k′ captures the effects of the game transitions. Even if cooperation is disfavored in each individual game, transitions between the games can be favorable for the evolution of cooperation. In fact, just slight differences between games can dramatically lower the barrier for the success of cooperators. Our results suggest that game transitions can play a critical role in the evolution of prosocial behaviors.

## Model

**Fig. 1.**
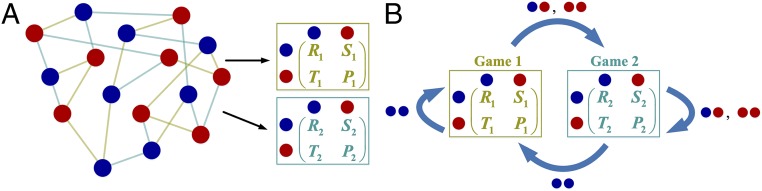
Game transitions on graphs. Each player occupies a node on the graph and has a strategic behavior, blue (“cooperate”) or red (“defect”), used in interactions with neighbors (*A*). In every time step, each player plays a game with every neighbor and accumulates its payoffs from all interactions. Games in different interactions can be different, highlighted by the color of edges and relevant payoff matrices. At the end of each time step, a random player is selected to be replaced, and all games update. Players’ behaviors and the game that they played in one time step determine the game to be played in the next time step (*B*). For example, if both players choose to take “red” behaviors in game 1 (i.e., mutual defection), they will play game 2 in the subsequent time step.

We study a population of N players consisting of cooperators, C, and defectors, D. The population structure is described by a graph. Each player occupies a node on the graph. Edges between nodes describe the events related to interactions and biological reproduction (or behavior imitation). In each time step, each player interacts separately with every neighbor, and the games played in different interactions can be distinct (Fig. 1*A*). When playing game i, mutual cooperation brings each player a “reward,” $R_{i}$, whereas mutual defection leads to an outcome of “punishment,” $P_{i}$; unilateral cooperation leads to a “sucker’s payoff,” $S_{i}$, for the cooperator and a “temptation,” $T_{i}$, for the defector. We assume that each game is a prisoner’s dilemma, which is defined by the payoff ranking $T_{i}>R_{i}>P_{i}>S_{i}$. Each player derives an accumulated payoff, π, from all interactions, and this payoff is translated into reproductive fitness, f=1−δ+δπ, where δ⩾0 represents the intensity of selection (24). We are particularly concerned with the effects of weak selection (25, 26), meaning that 0<δ≪1.

At the end of each time step, one player is selected for death uniformly at random from the population. The neighbors of this player then compete for the empty site, with each neighbor sending an offspring to this location with probability proportional to fitness. Following this “death–birth” update step, the games played in the population also update based on the previous games played and the actions taken in those games (Fig. 1*B*). For the player occupying the empty site, the games that it will play are determined by the interactions of the prior occupant.

The game transition can be deterministic or stochastic (probabilistic). If the game to be played is independent of the previous game, the game transition is “state independent” (18). When the game that will be played depends entirely on the previous game, the game transition is “behavior independent.” The simplest case is when the games in all interactions are identical initially and remain constant throughout the evolutionary process, which is the setup of most prior studies (6).

## Results

In the absence of mutation, a finite population will eventually reach a monomorphic state in which all players have the same strategy, either all cooperation or all defection. We study the competition between cooperation and defection by comparing the fixation probability of a single cooperator, $\rho_{C}$, with that of a single defector, $\rho_{D}$. Concretely, $\rho_{C}$ is the probability that a cooperator starting in a random location generates a lineage that takes over the entire population. Analogously, $\rho_{D}$ is the probability that a defector in a random position turns a population of cooperators into defectors. Selection favors cooperators relative to defectors if $\rho_{C}>\rho_{D}$ (24).

### Game Transitions between 2 States.

We begin with the case of deterministic game transitions between 2 states. Each state corresponds to a donation game (*SI Appendix*, sections 3 and 4 has a comprehensive investigation of 2-state games). In game 1, a cooperator bears a cost of c to bring its opponent a benefit of $b_{1}$, and a defector does nothing. Analogously, in game 2, a cooperator pays a cost of c to bring its opponent a benefit of $b_{2}$. That is, $R_{i}=b_{i}-c$, $S_{i}=-c$, $T_{i}=b_{i}$, and $P_{i}=0$ in game i. Both $b_{1}$ and $b_{2}$ are larger than c. The preferred choice for each player is defection, but $R_{i}>P_{i}$ in each game, resulting in the dilemma of cooperation. We say that game i is “more valuable” than game j if $b_{i}>b_{j}$. We take $b_{1}>b_{2}$ and explore a natural transition structure in which only mutual cooperation leads to the most valuable game.

**Fig. 2.**
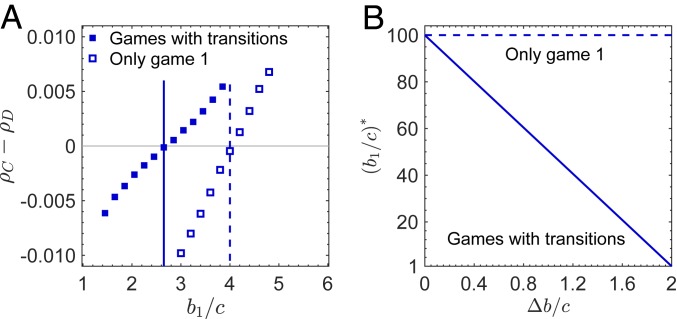
Game transitions can promote cooperation under death–birth updating. We study a transition structure between 2 donation games. A cooperator pays a cost c to bring its opponent a benefit $b_{1}$ in game 1 or $b_{2}$ in game 2; defectors pay nothing and provide no benefits. $b_{1}$ is larger than $b_{2}$. Mutual cooperation leads to game 1, and all other action profiles lead to game 2. Compared with only playing game 1, game transitions reduce the critical benefit-to-cost ratio, $\left(b_{1}/c\right)*$, for the evolution of cooperation (cross-points of dots and the horizontal line in *A*). Dots show simulation data, and vertical lines represent analytical results. Parameter values are N=500, k=4, δ=0.01, c=1, and $b_{2}=b_{1}-0.9$. In each simulation, all players play game 1 initially. Each simulation runs until the population reaches fixation (all C or all D), and each point is averaged over $10^{6}$ independent runs. A small difference between $b_{1}$ and $b_{2}$ ($\Delta b=b_{1}-b_{2}$) remarkably reduces the critical benefit-to-cost ratio $\left(b_{1}/c\right)*$ (*B*). We take k=100 in *B*.

If every player has k neighbors (i.e., the graph is “k regular”), we find that

$$\rho_{C}>\rho_{D}\Leftrightarrow \frac{b_{1}}{c}>k-\xi \frac{\Delta b}{c},$$ [1]

where $\Delta b=b_{1}-b_{2}$ and $\xi =\left(k-1\right)/2$. Note that ξ is positive and independent of payoff values, such as $b_{1}$, $b_{2}$, and c. We obtain this condition under weak selection based on the assumption that the population size N is much larger than k. When $b_{1}=b_{2}$, the 2 games are the same, which leads to the well-known rule of $b_{1}/c>k$ for cooperation to evolve on regular graphs (6). The existence of the term ξΔb/c indicates that transitions between different games can reduce the barrier for the success of cooperation. Even when both games oppose cooperation individually (i.e., $b_{1}/c<k$ and $b_{2}/c<k$), transitions between them can promote cooperation (Fig. 2*A*). Our analytical results agree well with numerical simulations.

The beneficial effects of game transitions on cooperation become more prominent on graphs of large degree, k. We find that a slight difference between games 1 and 2, Δb, can remarkably lower the barrier for cooperation to evolve. For example, when k=100 and c=1, the critical benefit-to-cost ratio, ${\left(b_{1}/c\right)}^{*}$, decreases from 100 to 50.5 for Δb=1.0 (Fig. 2*B*). Therefore, game transitions can significantly promote cooperation in realistic and highly connected societies (27). We find that similar results hold under the closely related “imitation” updating (*SI Appendix*, Fig. S1 and section 3).

**Fig. 3.**
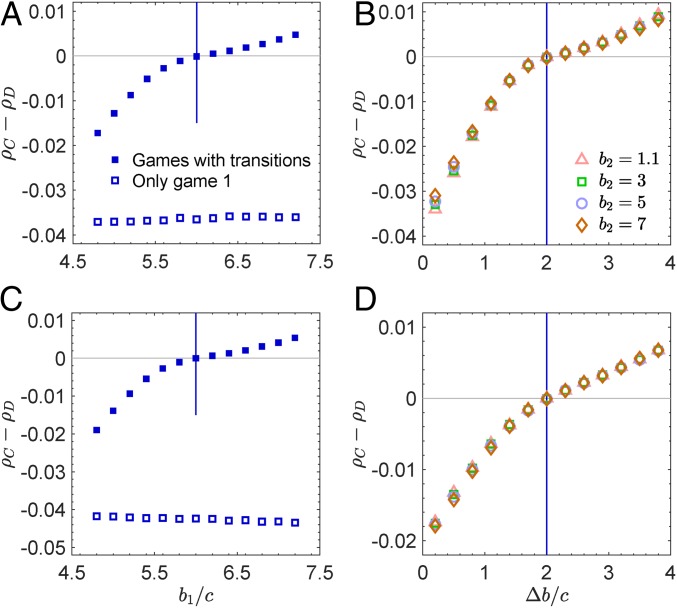
Game transitions can favor cooperation under birth–death (*A* and *B*) and pairwise-comparison updating (*C* and *D*). When individuals play only game 1, cooperation is disfavored over defection for any benefit-to-cost ratio, $b_{1}/c$ (*A* and *C*). When mutual cooperation leads to game 1 and other action profiles lead to game 2, cooperation can evolve. With game transitions, the difference between the 2 games, $\Delta b=b_{1}-b_{2}$, rather than the individual values of $b_{1}$ and $b_{2}$ determines the success of cooperation (*B* and *D*). Parameter values are N=500, k=4, δ=0.01, c=1, and $b_{2}=4$ (*A* and *C*). In each simulation, all players play game 1 initially. Each simulation runs until the population reaches fixation, and each point is averaged over $10^{6}$ independent runs.

Next, we consider “birth–death” (28) and “pairwise-comparison” (29, 30) updating. Under birth–death updating, in each time step, a random player is selected for reproduction with probability proportional to fitness. The offspring replaces a random neighbor. Under pairwise-comparison updating, a player is first selected uniformly at random to update his or her strategy. When player i is chosen for a strategy updating, it randomly chooses a neighbor j and compares payoffs. If $\pi_{i}$ and $\pi_{j}$ are the payoffs to i and j, respectively, player i adopts j’s strategy with probability $1/\left[1+\exp\left(-\delta \left(\pi_{j}-\pi_{i}\right)\right)\right]$ and retains its old strategy otherwise. When mutual cooperation leads to game 1 and other action profiles lead to game 2, under both birth–death and pairwise-comparison updating, we have the rule

$$\rho_{C}>\rho_{D}\Leftrightarrow \xi \frac{\Delta b}{c}>1,$$ [2]

where ξ=1/2 (*SI Appendix*, sections 3 and 4). When the 2 games are the same, Δb=0, and cooperators are never favored over defectors (Fig. 3 *A* and *C*). Game transitions provide an opportunity for cooperation to thrive as long as $b_{1}-b_{2}>c/\xi$, which opens an avenue for the evolution of cooperation under birth–death and pairwise-comparison updating. One can attribute this result to the fact that, under this transition structure, mutual cooperation results in $b_{1}-c$, but when 2 players use different actions, the cooperator gets −c and the defector gets $b_{2}$. If $b_{1}-b_{2}>c/\xi$, then it must be true that $b_{1}-c>b_{2}$, which means that the players are effectively in a coordination game with a preferred outcome of mutual cooperation.

More intriguingly, Eq. **2** shows that the success of cooperators depends on the difference between benefits provided by an altruistic behavior in game 1 and game 2, and it is independent of the exact value in each game (Fig. 3 *B* and *D*). Thus, in a dense population where individuals have many neighbors, even if the benefits provided by an altruistic behavior are low in both game 1 and game 2, transitions between them can still support the evolution of cooperation. We stress that the difference between the 2 games required to favor cooperation is surprisingly small. For example, $b_{1}-b_{2}>2c$ warrants the success of cooperation over defection on graphs of any degree.

We further examine random graphs (31) and scale-free networks (32), where players differ in the number of their neighbors (*SI Appendix*, Fig. S2). We find that game transitions can provide more advantages for the evolution of cooperation than their static counterparts under death–birth and imitation updating, and they also give a way for cooperation to evolve under birth–death and pairwise-comparison updating. In addition, we study evolutionary processes with mutation and/or behavior exploration (*SI Appendix*, Fig. S3). The results demonstrate the robustness of the effects of game transitions on the evolution of cooperation.

### Stochastic, State-Independent Transitions.

For more general state-independent transitions between 2 games, let p and q represent the probabilities of transitioning to game 2 (the less-valuable game) after mutual cooperation and after unilateral cooperation/defection, respectively. Under death–birth updating, the condition for cooperation to be favored over defection follows the format of Eq. **1** with

$$\xi =\frac{k-1}{2}q-\frac{k+1}{2}p.$$ [3]

**Fig. 4.**
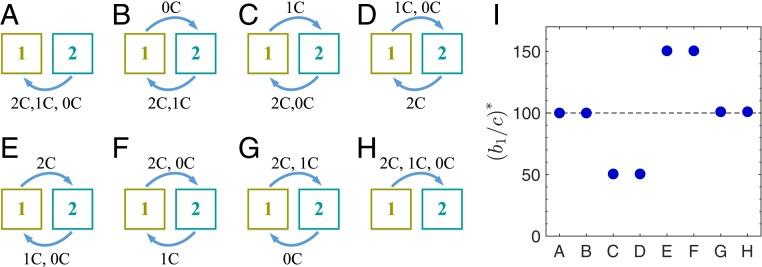
Critical ratio for the evolution of cooperation as a function of the game transition pattern. Game transitions are state independent, which means that the game to be played in the next time step depends on only the number of cooperators. For $\Delta b_{12}=1$ and k=100, we calculate the threshold $\left(b_{1}/c\right)*$ (I) for all 8 deterministic transitions between 2 states (*A–H*). Game transitions promote cooperation only when mutual cooperation always allows for a more valuable game 1 and unilateral defection leads to a less valuable game 2 (*C* and *D*). The transition after mutual cooperation or unilateral defection is critical to the evolutionary outcome. For example, modifying the transition responding to mutual cooperation (*B* and *F*) or unilateral defection (*B* and *D*) changes $\left(b_{1}/c\right)*$ significantly. However, the transitions responding to mutual defection have negligible effects. Critical ratios, $\left(b_{1}/c\right)*$: 100 (*A* and *B*), 50.5 (*C* and *D*), 150.5 (*E* and *F*), and 101 (*G* and *H*).

The example in Fig. 2 corresponds to p=0 and q=1. We explore all 8 deterministic game transitions in Fig. 4. We see that game transitions promote cooperation only when mutual cooperation leads to a more profitable game 1 and unilateral defection leads to a less profitable game 2 (Fig. 4 *C* and *D*). However, when mutual cooperation leads to a detrimental state 1 and unilateral defection leads to a beneficial state 2, it is more difficult for cooperation to evolve (Fig. 4 *E* and *F*). In particular, whether or not the game transitions affect the evolution of cooperation depends strongly on the transition after mutual cooperation and the transition after unilateral cooperation/defection. For example, in Fig. 4 *B* and *F*, changing the transition following mutual cooperation influences ${\left(b_{1}/c\right)}^{*}$ considerably. Transitions following mutual defection play a less prominent role (Fig. 4 *C* and *D*).

### Game Transitions among n States.

We turn now to the general setup of game transitions among n states (i.e., games 1 through n). If 2 players play game i in the current time step and among them there are $s\in \left\{0,1,2\right\}$ cooperators, they will play game j in the next time step with probability $p_{ij}^{\left(s\right)}$. s is 2 for mutual cooperation, 1 for unilateral cooperation/defection, and 0 for mutual defection. In the prior example of the state transitioning to game 1 after mutual cooperation and to game 2 otherwise, we have n=2 and $p_{21}^{\left(2\right)}=1$. This setup can recover deterministic or probabilistic transitions, state dependent or independent, behavior dependent or independent, and the traditional models involving only a single game (6, 13) as specific cases. We assume that all games are donation games (*SI Appendix*, section 3 discusses any 2-player, 2-strategy game). In game i, a cooperator pays a cost of c to bring its opponent a benefit of $b_{i}$. Game 1 is the most valuable, meaning that $b_{1}⩾b_{i}$ for every i.

Under death–birth updating, we find that

$$\rho_{C}>\rho_{D}\Leftrightarrow \frac{b_{1}}{c}>k-\overset{n}{\underset{i=2}{\sum }}\xi_{i}\frac{\Delta b_{1i}}{c},$$ [4]

where for every i, $\Delta b_{1i}=b_{1}-b_{i}$, and $\xi_{i}$ depends on the game transition pattern [i.e., $p_{ij}^{\left(s\right)}$] but is independent of the benefit in each game, $b_{i}$, and cost, c (*Calculation of* $\xi_{i}$ discusses the calculation of $\xi_{i}$). The term $\sum_{i=2}^{n}\xi_{i}\Delta b_{1i}/c$ captures how game transitions influence this threshold. The effects of game transitions on cooperation actually arise from 2 sources: the game transition pattern and the variation in different games. $\xi_{i}$ captures the former, and $\Delta b_{1i}/c$ captures the latter.

Importantly, these 2 components are independent, which makes it easier to understand the role of each. Let k′ denote $\sum_{i=2}^{n}\xi_{i}\Delta b_{1i}/c$, and let b denote $b_{1}$. We can interpret Eq. **4** intuitively: weak selection favors cooperation if the ratio of the benefit from an altruistic behavior, b, to its cost, c, exceeds the average effective number of neighbors, k−k′. Analogously, under birth–death or pairwise-comparison updating, we find that

$$\rho_{C}>\rho_{D}\Leftrightarrow \overset{n}{\underset{i=2}{\sum }}\xi_{i}\frac{\Delta b_{1i}}{c}>1.$$ [5]

We refer the reader to *Calculation of* $\xi_{i}$ for the calculation of $\xi_{i}$.

Our study above assumes that, in each time step, games played by any 2 players are likely to update (“global” transitions). We also consider the case that games in only a fraction of interactions have chance to update. When games to be updated are randomly selected from the whole population, such a game transition can be transformed to the global transition with a modified transition matrix (*SI Appendix*, section 3). Therefore, Eqs. **4** and **5** still predict the evolutionary outcome.

We also study the case in which the games to be updated are spatially correlated, with only those nearby an individual who competes to reproduce being affected (“local” transitions). Under death–birth and pairwise-comparison updating, global and local transitions lead to decidedly different models. We show that, however, the simple rules for cooperation to evolve (Eqs. **4** and **5**) still hold provided that $\xi_{i}$ is modified (*SI Appendix*, Figs. S4 and S5, and section 1). We give a brief overview of local game transitions in *Global vs. Local Game Transitions*.

## Pure vs. Stochastic Strategies

So far, in every time step, each player is either a cooperator or a defector. However, the model that we propose here has a much broader scope than just 2 pure strategies. For example, we also investigate the competition between stochastic strategies under game transitions. Let $s_{p}$ denote a stochastic strategy with which, in each time step, a player chooses cooperation with probability p and defects otherwise. $s_{1}$ thus corresponds to a pure cooperator, and $s_{0}$ corresponds to a pure defector.

We find that the condition for $s_{p}$ being favored by selection over $s_{q}$ still follows the format of Eq. **4** under death–birth updating and Eq. **5** under birth–death or pairwise-comparison updating, provided that $\xi_{i}$ is modified (*SI Appendix*, section 3). When mutual cooperation leads to a more valuable game and other action profiles lead to a less valuable game, under death–birth updating, game transitions lower the threshold for a cooperative strategy (i.e., $s_{p}$ with a large p) being favored relative to a less cooperative strategy. We also find that game transitions can favor the evolution of a cooperative stochastic strategy under birth–death and pairwise-comparison updating.

## Discussion

We consider evolutionary dynamics with game transitions, coupling individuals’ actions with the environment. Individuals’ behaviors modify the environment, which in turn, affects the viability of future actions in that environment. We find a simple rule for the success of cooperators in an environment that can switch between an arbitrary number of states, namely b/c>k−k′, where k′ exactly captures how game transitions affect the evolution of cooperation. When all environmental states are identical, we recover the rule b/c>k (6).

In a 2-action game governed by a single payoff matrix with entries R, S, T, and P, the so-called “sigma rule” of Tarnita et al. (33) says that there exists σ for which cooperators are favored over defectors if and only if σR+S>T+σP. The coefficient σ, which is independent of the payoffs, captures how the spatial model and its associated update rule affect evolutionary dynamics. For an infinite random regular graph under death–birth updating, $\sigma =\left(k+1\right)/\left(k-1\right)$. When all interactions are governed by a donation game with a donation cost c and benefit $b_{1}$, substituting $R=b_{1}-c$, S=−c, $T=b_{1}$, and P=0 into the sigma rule gives the condition of cooperation being favored over defection. Intriguingly, Eq. **1** can be phrased in the form of a sigma rule, with $R=b_{1}-c+\left(b_{1}-b_{2}\right)\left(k-1\right)/\left(k+1\right)$, S=−c, $T=b_{1}$, and P=0. With game transitions, evolution proceeds “as if” all interactions are governed by an effective game with $R=b_{1}-c+\left(b_{1}-b_{2}\right)\left(k-1\right)/\left(k+1\right)$, S=−c, $T=b_{1}$, and P=0. Compared with the donation game, mutual cooperation brings each player an extra benefit of $\left(b_{1}-b_{2}\right)\left(k-1\right)/\left(k+1\right)$ in this effective game. That is, the game transitions create a situation in which 2 cooperators play a synergistic game and obtain synergistic benefits (more discussions are in *SI Appendix*, section 3*E*).

This intuition also holds for birth–death and pairwise-comparison updating. For a prisoner’s dilemma in a constant environment, weak selection disfavors cooperation in any homogeneous structured population (6, 34). With game transitions, the synergistic benefit to each cooperator on their mutual cooperation induces a transformation of the payoff structure. In particular, the synergistic benefit can transform the nature of the interaction from a prisoner’s dilemma to a coordination game with a preferred outcome of mutual cooperation.

The fact that game transitions allow cooperation to evolve is related to the idea of partner-fidelity feedback in evolutionary biology (35, 36). Partner-fidelity feedback describes that one’s cooperation increases its partner’s fitness, which ultimately, feeds back as a fitness increase to the cooperator. Unlike reactive strategies like Tit-for-Tat, this feedback is an automatic process and does not require the partner’s conditional response. In the classic example of grass-endophyte mutualism (37, 38), by producing secondary compounds to protect the grass host, endophytes obtain more nutritional provisioning from the host. By providing nutrients to the endophytes, the grass host is more resistant to herbivores due to the increased delivery of secondary compounds. Similarly, in our study, mutual cooperation could generate a synergistic benefit, which in turn, promotes the evolution of cooperation.

When mutual cooperation allows for a more profitable game and other actions profiles lead to a less profitable game, a slight difference between games considerably reduces the threshold for the evolution of cooperation. The reason is that, although the variation in games might be orders of magnitude smaller than the threshold for establishing cooperation, transitions among such games generate a synergistic benefit on mutual cooperation that is of the same order of magnitude as the cost of a cooperative act. Since the synergistic benefit partly makes up for the loss from a cooperative act, a slight difference between games makes cooperation less costly. This finding is of significance to understanding large-scale cooperation in many highly connected social networks. In these networks, an individual can have hundreds of neighbors (27, 39), and cooperators thus face the risk of being exploited by many neighboring defectors. If the environment remains constant, cooperation must be profitable enough to make up for exploitation by defection (6). Game transitions can act to reduce the threshold for maintaining cooperation considerably.

We also find that game transitions can stabilize cooperation even when mutation or random strategy exploration is allowed. In a constant environment, when a mutant defector arises within a cluster of cooperators, it dilutes the spatial assortment of cooperators and thus, hinders the evolution of cooperation (40). When the environment changes as a result of individuals’ behaviors, although the defecting mutant indeed exploits its neighboring cooperators temporarily, the environment in which this happens deteriorates rapidly. As a result, the temptation to defect is weakened. In a constant environment, selection also favors the establishment of spatial assortment, whereas mutation destroys it continuously. The population state finally reaches a “mutation-selection” stationary distribution. However, when the environment is subject to transitions, the interaction environment would also be a part of this distribution. In this case, the joint distribution over individuals’ states and games could be described as a “game-mutation-selection” stationary distribution.

Recent years have seen a growing interest in exploring evolutionary dynamics in a changing and/or heterogeneous environments (41–50). Our model is somewhat different. Our study accounts for both exogenous factors and individuals’ behaviors in the change of the environment, modeling general environmental feedback. In addition, the environment that 2 players face is independent of that of another pair of players. Individuals’ strategic behaviors directly influence the environment in which they evolve, which enables an individual to reciprocate with the opponent in a single interaction through environmental feedback. Therefore, even if cooperators are disfavored in each individual environment, cooperators can still be favored over defectors through environmental reciprocity. Such an effect has never been observed in prior studies where all individuals interact in a homogeneous environment (41, 44). In those studies, although the environments that individuals face are different, at any specific stage the environment is identical for all individuals. When defection is a dominant strategy in each individual environment, defection also dominates cooperation in the context of an ever-changing environment (41, 42, 44). In a recent work, Hilbe et al. (18) found that individuals can rely on repeated interactions and continuous strategies to achieve environmental reciprocity. Compared with their model, in our setup, individuals play a one-shot game with a pure, unconditional strategy. Our model shows that, without relying on direct reciprocity and any strategic complexity, game transitions can still promote the evolution of cooperation.

### Data Availability Statement.

There are no associated data.

## Calculation of $\xi_{i}$

Let $p_{ij}^{\left(s\right)}$ (i,j∈{1,2,…,n} and $s\in \left\{0,1,2\right\}$) be the probability that the state transitions from game i to game j after s players cooperate. Let $P^{\left(s\right)}$ denote a game transition matrix, where $p_{ij}^{\left(s\right)}$ is the element in the ith row and jth column. We present the formula of $\xi_{i}$ for a class of game transition patterns here and show the calculation of ξ for general transitions in *SI Appendix*, section 3.

For every $s\in \left\{0,1,2\right\}$, suppose that the Markov chain with state space $\left\{1,2,\ldots ,n\right\}$ and transition matrix $P^{\left(s\right)}$ has only one recurrence class (and that the states therein are aperiodic). Let $u^{\left(s\right)}=\left(u_{1}^{\left(s\right)},\ldots ,u_{n}^{\left(s\right)}\right)$ denote the stationary distribution of this chain: that is, the solution to $u^{\left(s\right)}=u^{\left(s\right)}P^{\left(s\right)}$ with $\sum_{j=1}^{n}u_{i}^{\left(s\right)}=1$. We have (*SI Appendix*, section 3)

$$\xi_{i}=\frac{\left(k-1\right)}{2}u_{i}^{\left(1\right)}-\frac{\left(k+1\right)}{2}u_{i}^{\left(2\right)}$$

for death–birth updating and

$$\xi_{i}=\frac{u_{i}^{\left(1\right)}}{2}-\frac{u_{i}^{\left(2\right)}}{2}$$

for birth–death or pairwise-comparison updating. In particular, for game transitions between 2 states, we have

$$\xi_{2}=\frac{\left(k-1\right)p_{12}^{\left(1\right)}}{2\left(p_{12}^{\left(1\right)}+p_{21}^{\left(1\right)}\right)}-\frac{\left(k+1\right)p_{12}^{\left(2\right)}}{2\left(p_{12}^{\left(2\right)}+p_{21}^{\left(2\right)}\right)}$$

for death–birth updating and

$$\xi_{2}=\frac{p_{12}^{\left(1\right)}}{2\left(p_{12}^{\left(1\right)}+p_{21}^{\left(1\right)}\right)}-\frac{p_{12}^{\left(2\right)}}{2\left(p_{12}^{\left(2\right)}+p_{21}^{\left(2\right)}\right)}$$

for birth–death or pairwise-comparison updating. For other game transitions, the evolutionary dynamics (and thus, $\xi_{i}$) may be sensitive to the initial condition (i.e., the initial fractions of various games). We illustrate an example calculation of $\xi_{i}$ in *SI Appendix*, section 4.

## Global vs. Local Game Transitions

**Fig. 5.**
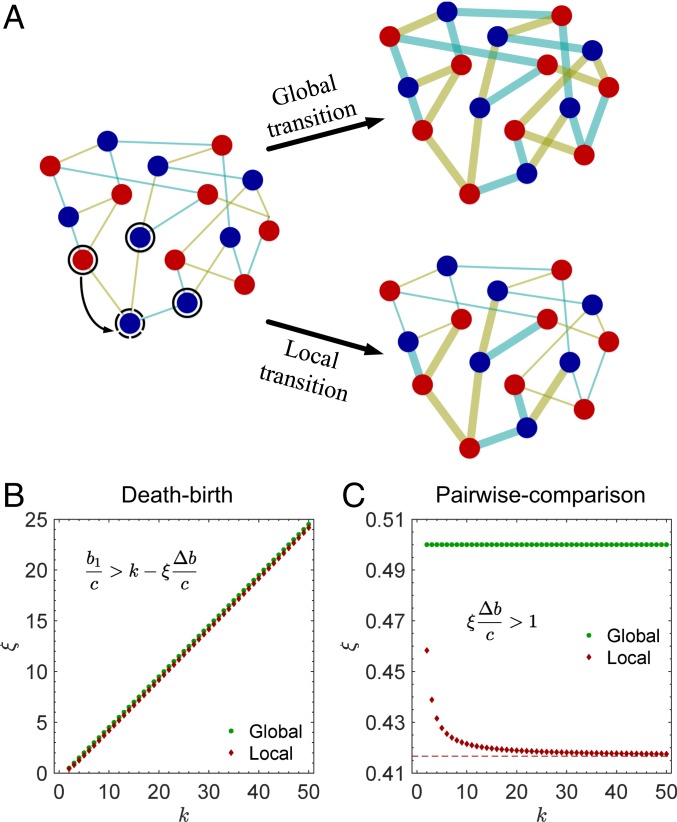
Global and local game transitions. Depicted in *A* is an example of game transitions in one time step under death–birth updating. A random player (dashed circle) is chosen for death; subsequently, this individual’s neighbors (solid circles) compete to reproduce and send an offspring into the vacancy with a probability proportional to fitness. With global game transitions, games in all interactions update in each time step. With local game transitions, only the games involved with players that compete to reproduce update (depicted by bold edges). We examine both global and local transitions under death–birth (*B*) and pairwise-comparison updating (*C*). When a game has an opportunity to update, it transitions to a more valuable game 1 after mutual cooperation and to a less valuable game 2 after defection. Game transitions, regardless of whether they are global or local, can promote cooperation markedly, although in this case, global transitions result in a more relaxed condition for the evolution of cooperation than do local transitions.

Our study above assumes that game transitions are an automatic (and exogenous) responses to interactions. Thus, in each time step, the games played by any 2 players are likely to update (global transitions). However, when the game transitions are subject to individuals’ willingness to play a game, players could present different tendencies to modify the environments in which they evolve. For example, under death–birth updating, if player i is selected for death, then only i’s nearest neighbors compete to reproduce and replace i with an offspring. Compared with those not involved in competition around the vacant site, individuals close to the individual to be replaced have stronger incentives to change the environment that they face, since this environment affects their success in filling the vacancy. In other words, games induced by the nearest neighbors of the deceased drive the evolution of a system. Therefore, one could impose transitions only on these games, leading to local transitions (Fig. 5*A*).

Birth–death updating requires competition at the population level, and therefore, global and local transitions are identical in this case. For death–birth and pairwise-comparison updating, however, global and local transitions lead to different models. We show that the simple rules for cooperation to evolve (Eqs. **4** and **5**) still hold provided that $\xi_{i}$ is modified (*SI Appendix*, sections 1 and 4). Specifically, we consider the following transition pattern: when a game has an opportunity to update, it transitions to a more valuable game 1 after mutual cooperation and to a less valuable game 2 after defection. Under death–birth updating, we have $\rho_{C}>\rho_{D}$ if and only if $b_{1}/c>k-\xi \Delta b/c$, where $\xi =\left(6k^{4}-10k^{3}+3k^{2}+6k+2\right)/\left(12k^{3}\right)$ (compared with ξ=(k−1)/2 for global transitions). For pairwise-comparison updating, $\rho_{C}>\rho_{D}$ if and only if ξΔb/c>1, where $\xi =\left(10k^{2}-4k+1\right)/\left(24k^{2}-12k\right)$ (compared with ξ=1/2 for global transitions).

According to the nature of the critical threshold ($b_{1}/c>k-\xi \Delta b/c$ for death–birth updating and ξΔb/c>1 for pairwise-comparison updating), global transitions act as a more effective promoter of cooperation than local transitions do (Fig. 5 *B* and *C*). However, for both kinds of game transitions, many messages are qualitatively the same: game transitions can promote cooperation (Figs. 2 and 3 and *SI Appendix*, Fig. S4), game transitions can amplify the beneficial effects of game variations on cooperation (Figs. 2 and 3 and *SI Appendix*, Fig. S5), and game transitions responding to mutual cooperation or unilateral cooperation/defection strongly affect cooperation. We include a more detailed discussion of global vs. local game transitions in *SI Appendix*, section 4.

## Supplementary Material

Supplementary File
