# Supplementary material for: Evolutionary dynamics with game transitions
Source: Proc Natl Acad Sci U S A. 2019 Nov 26;116(51):25398–404. doi: 10.1073/pnas.1908936116 (PMC6926053; doi:10.1073/pnas.1908936116)
Supplement: Supplementary File [file pnas.1908936116.sapp.pdf]

Supplementary Information for

Evolutionary dynamics with game transitions

Qi Su, Alex McAvoy, Long Wang, and Martin A. Nowak

Alex McAvoy, Long Wang, Martin A. Nowak.
E-mail: alexmcavoy@fas.harvard.edu, longwang@pku.edu.cn, martin_nowak@harvard.edu

This PDF file includes:

Supplementary text
Figs. S1 to S5
References for SI reference citations

www.pnas.org/cgi/doi/10.1073/pnas.1908936116

Supporting Information Text

The supplementary information is structured as follows.

In Section 1, we study evolutionary dynamics with local game transitions. We derive an analytical condition for one strategy to be favored over the other. A further analysis gives the mathematical formula for the critical benefit-to-cost ratio for cooperation to evolve.

In Section 2, we study how the initial condition (initial fractions of various games played in the population) affects the evolutionary outcomes. We provide an effective approach to evaluate whether or not the evolutionary dynamics is sensitive to the initial condition.

In Section 3, we study evolutionary dynamics with global game transitions. We derive an analytical condition of one strategy to be favored over the other, as well as the critical benefit-to-cost ratio for cooperation to evolve. We prove that our rules also hold when players use stochastic strategies (cooperate or defect with a probability rather than unconditionally).

In Section 4, we study four representative examples, including state-independent game transitions (the game to be played is independent of games played in the past), strategy-independent game transitions (the game to be played is independent of players' actions in the past), game transitions between two states (including the example presented in the main text), and probabilistic game transitions between three states (transitions between different games with a probability). We show that how probabilistic game transitions affect the favorable effects of game transitions on cooperation may depend on the variations in different games.

1. Evolutionary dynamics with local game transitions

We consider game transitions among n states, described by games $1, 2, \ldots, n$. The payoff structure of game i is

$$\begin{array}{c} \\ A \\ B \end{array} \begin{array}{c} \begin{array}{cc} A & B \end{array} \\ \begin{pmatrix} R_i & S_i \\ T_i & P_i \end{pmatrix} \end{array}, \qquad [1]$$

where each value corresponds to a payoff derived by a player with a strategy in the column against a player with a strategy in the row. The game transition pattern is described by three matrices, i.e.

$$\mathbf{P}^{(2)} = \begin{bmatrix} p_{11}^{(2)} & \cdots & p_{1n}^{(2)} \\ \vdots & \ddots & \vdots \\ p_{n1}^{(2)} & \cdots & p_{nn}^{(2)} \end{bmatrix}, \quad \mathbf{P}^{(1)} = \begin{bmatrix} p_{11}^{(1)} & \cdots & p_{1n}^{(1)} \\ \vdots & \ddots & \vdots \\ p_{n1}^{(1)} & \cdots & p_{nn}^{(1)} \end{bmatrix}, \quad \mathbf{P}^{(0)} = \begin{bmatrix} p_{11}^{(0)} & \cdots & p_{1n}^{(0)} \\ \vdots & \ddots & \vdots \\ p_{n1}^{(0)} & \cdots & p_{nn}^{(0)} \end{bmatrix}, \qquad [2]$$

where $p_{ij}^{(s)}$ represents the probability that players play game j in the next time step conditioned on that they play game i in the current time step and there are s A-players, where $i, j \in \{1, 2, \ldots, n\}$ and $s \in \{0, 1, 2\}$.

On graphs or social networks, each player occupies a node. If two players (or nodes occupied by players) are connected by an edge or a social tie, they play a one-shot game in each time step. The main idea of the theoretical analysis is to couple the game played by two connected players and their strategy profiles into edges. Let $E_{XY}^{(i)}$ denote an edge in which the two connected players take strategies X and Y ($X, Y \in \{A, B\}$), respectively, in game $i \in \{1, 2, \ldots, n\}$. For example, in edge $E_{AA}^{(1)}$, both of the players take A strategy and they play game 1. We then introduce the following variables to describe this evolving system:
p_A: the frequency of A-players;
p_B: the frequency of B-players;
$p_{XY}^{(i)}$: the frequency of edge $E_{XY}^{(i)}$;
$q_{X|Y}^{(i)}$: the probability to find an edge $E_{XY}^{(i)}$ given that one node of this edge is occupied by a $Y-$player;
p_{XY}: the frequency of edges that connect an X-player and a Y-player;
$q_{X|Y}$: the conditional probability to find an $X-$player given that the adjacent node is occupied by a $Y-$player.

Then we have the identities

$$p_A + p_B = 1; \qquad [3a]$$

$$\sum_{i=1}^{n} q_{X|Y}^{(i)} = q_{X|Y}; \qquad [3b]$$

$$p_{XY}^{(i)} = q_{X|Y}^{(i)} p_Y; \qquad [3c]$$

$$p^{(i)}_{XY} = p^{(i)}_{YX}; \qquad [3d]$$

$$q_{A|X} + q_{B|X} = 1; \qquad [3e]$$

$$p_{XY} = q_{X|Y} p_Y. \qquad [3f]$$

Note that players' strategies and the game they play coevolve throughout the evolutionary process. From the perspective of network dynamics, we need to consider the change in the frequency of nodes occupied by A-players and the frequency of edge $E^{(i)}_{XY}$. Based on above identities, we can use p_A and $q^{(i)}_{X|Y}$ to describe the whole system. In the following, we study a random regular graph, where each node is linked to other k nodes.

A. Interactions. In each time step, each player interacts separately with every neighbor, and the games played in different interactions can be distinct. Each player derives an accumulated payoff, π, from all interactions, and this payoff is translated into reproductive fitness, $f = 1 - \delta + \delta\pi$, where $\delta \geqslant 0$ represents the intensity of selection (1). δ scales the contribution of games played to one's fitness/reproductive rates. The assumption of $\delta \ll 1$, termed weak selection, describes that the game plays only a very small role or it represents only one of many factors influencing the overall reproductive rate. Besides, this assumption allows to derive analytical results and has been widely used in evolutionary biology (1, 2). We focus on the effects of weak selection (3, 4).

B. Death-birth updating. Under death-birth updating, in each time step, a random player is selected to die; all neighbors then compete to reproduce and send an offspring to the vacant site (with the probability proportional to fitness) (5). We can also interpret this updat rule in a social setting: a random player i decides to update his or her strategy; subsequently, he or she adopts a neighbor's strategy with a probability proportional to the neighbor's fitness. Local transitions account for the fact that only the nearest neighbors compete for the vacancy. When the environment change is subject to human's willingness, these neighbors, compared with other players not involved in the competition, are more incentivized to modify the environment (games) in which they evolve. Therefore, under local game transitions, only games played by the nearest neighbors of the dead can update. We first investigate the change in the frequency of $A-$players.

B.1. Change in p_A—updating a B-player. A B-player is chosen to die with probability p_B. Let $k^{(i)}_{A|B}$ denote the number of neighbors who adopt strategy A and play game i with the focal (dead) player. Analogously, $k^{(i)}_{B|B}$ denotes the number of neighbors who adopt strategy B and play game i with the focal player. Therefore, $\sum_{i=1}^{n}\left(k^{(i)}_{A|B} + k^{(i)}_{B|B}\right) = k$. The probability for such a neighborhood configuration is

$$\mathcal{B}\left(k^{(i)}_{A|B}, k^{(i)}_{B|B} | i = 1, \ldots, n\right) = \frac{k!}{\Pi_{i=1}^{n}\left(k^{(i)}_{A|B}! k^{(i)}_{B|B}!\right)} \Pi_{i=1}^{n}\left[\left(q^{(i)}_{A|B}\right)^{k^{(i)}_{A|B}} \left(q^{(i)}_{B|B}\right)^{k^{(i)}_{B|B}}\right]. \qquad [4]$$

The fitness of a neighbor who adopts strategy A and plays game i with the focal player is

$$f^{(i)}_{A|B} = 1 - \delta + \delta\left[(k-1)\sum_{j=1}^{n} q^{(j)}_{A|A} R_j + (k-1)\sum_{j=1}^{n} q^{(j)}_{B|A} S_j + S_i\right]. \qquad [5]$$

The fitness of a neighbor who adopts strategy B and plays game i with the focal player is

$$f^{(i)}_{B|B} = 1 - \delta + \delta\left[(k-1)\sum_{j=1}^{n} q^{(j)}_{A|B} T_j + (k-1)\sum_{j=1}^{n} q^{(j)}_{B|B} P_j + P_i\right]. \qquad [6]$$

The probability that one of neighboring A-players replaces the vacancy under such a neighborhood configuration is given by

$$\mathbb{P}(A \to B) = \frac{\sum_{i=1}^{n} k^{(i)}_{A|B} f^{(i)}_{A|B}}{\sum_{i=1}^{n}\left(k^{(i)}_{A|B} f^{(i)}_{A|B} + k^{(i)}_{B|B} f^{(i)}_{B|B}\right)}. \qquad [7]$$

The probability that one of neighboring $B-$players replaces the vacancy under such a neighborhood configuration is given by

$$\mathbb{P}(B \to B) = \frac{\sum_{i=1}^{n} k^{(i)}_{B|B} f^{(i)}_{B|B}}{\sum_{i=1}^{n}\left(k^{(i)}_{A|B} f^{(i)}_{A|B} + k^{(i)}_{B|B} f^{(i)}_{B|B}\right)}. \qquad [8]$$

Therefore, p_A increases by $1/N$ with probability

$$\mathbb{P}\left(\Delta p_A = \frac{1}{N}\right) = p_B \sum_{\sum_{i=1}^{n}\left(k_{A|B}^{(i)}+k_{B|B}^{(i)}\right)=k} \mathcal{B}\left(k_{A|B}^{(i)}, k_{B|B}^{(i)}|i=1,\ldots,n\right)\mathbb{P}\left(A\to B\right). \quad [9]$$

B.2. Change in p_A—updating a A-player. An A-player is chosen to die with probability p_A. Let $k_{A|A}^{(i)}$ denote the number of neighbors who adopt strategy A and play game i with the focal player. Analogously, $k_{B|A}^{(i)}$ denotes the number of neighbors who adopt strategy B and play game i with the focal player. Therefore, $\sum_{i=1}^{n}\left(k_{A|A}^{(i)}+k_{B|A}^{(i)}\right)=k$. The probability for such a neighborhood configuration is

$$\mathcal{A}\left(k_{A|A}^{(i)}, k_{B|A}^{(i)}|i=1,\ldots,n\right) = \frac{k!}{\Pi_{i=1}^{n}\left(k_{A|A}^{(i)}!k_{B|A}^{(i)}!\right)}\Pi_{i=1}^{n}\left[\left(q_{A|A}^{(i)}\right)^{k_{A|A}^{(i)}}\left(q_{B|A}^{(i)}\right)^{k_{B|A}^{(i)}}\right]. \quad [10]$$

The fitness of a neighbor who adopts strategy A and plays game i with the focal player is

$$f_{A|A}^{(i)} = 1-\delta+\delta\left[(k-1)\sum_{j=1}^{n} q_{A|A}^{(j)}R_j + (k-1)\sum_{j=1}^{n} q_{B|A}^{(j)}S_j + R_i\right]. \quad [11]$$

The fitness of a neighbor who adopts strategy B and plays game i with the focal player is

$$f_{B|A}^{(i)} = 1-\delta+\delta\left[(k-1)\sum_{j=1}^{n} q_{A|B}^{(j)}T_j + (k-1)\sum_{j=1}^{n} q_{B|B}^{(j)}P_j + T_i\right]. \quad [12]$$

The probability that one of neighboring A-players replaces the vacancy under such a neighborhood configuration is given by

$$\mathbb{P}\left(A\to A\right) = \frac{\sum_{i=1}^{n} k_{A|A}^{(i)}f_{A|A}^{(i)}}{\sum_{i=1}^{n}\left(k_{A|A}^{(i)}f_{A|A}^{(i)}+k_{B|A}^{(i)}f_{B|A}^{(i)}\right)}. \quad [13]$$

The probability that one of neighboring $B-$players replaces the vacancy under such a neighborhood configuration is given by

$$\mathbb{P}\left(B\to A\right) = \frac{\sum_{i=1}^{n} k_{B|A}^{(i)}f_{B|A}^{(i)}}{\sum_{i=1}^{n}\left(k_{A|A}^{(i)}f_{A|A}^{(i)}+k_{B|A}^{(i)}f_{B|A}^{(i)}\right)}. \quad [14]$$

Therefore, p_A decreases by $1/N$ with probability

$$\mathbb{P}\left(\Delta p_A = -\frac{1}{N}\right) = p_A \sum_{\sum_{i=1}^{n}\left(k_{A|A}^{(i)}+k_{B|A}^{(i)}\right)=k} \mathcal{A}\left(k_{A|A}^{(i)}, k_{B|A}^{(i)}|i=1,\ldots,n\right)\mathbb{P}\left(B\to A\right). \quad [15]$$

B.3. Change in p_A. Let us now suppose that one strategy replacement event takes place in one unit of time. The time derivative of p_A is given by

$$\begin{aligned}\dot{p}_A &= \frac{1}{N}\mathbb{P}\left(\Delta p_A = \frac{1}{N}\right) + \left(-\frac{1}{N}\right)\mathbb{P}\left(\Delta p_A = -\frac{1}{N}\right)\\ &= \delta\frac{1}{kN}\sum_{i=1}^{n}\left(I_{R_i}R_i + I_{S_i}S_i + I_{T_i}T_i + I_{P_i}P_i\right) + O\left(\delta^2\right),\end{aligned} \quad [16]$$

where

$$I_{R_i} = p_{AA}^{(i)}(k-1)\left[(k-1)q_{B|A}(q_{A|A}+q_{B|B})+q_{B|A}\right]; \quad [17a]$$

$$I_{S_i} = p_{AB}^{(i)}(k-1)\left[(k-1)q_{B|A}(q_{A|A}+q_{B|B})+q_{B|B}\right]; \quad [17b]$$

$$I_{T_i} = -p_{AB}^{(i)}(k-1)\left[(k-1)q_{A|B}(q_{A|A}+q_{B|B})+q_{A|A}\right]; \quad [17c]$$

$$I_{P_i} = -p_{BB}^{(i)}(k-1)\left[(k-1)q_{A|B}(q_{A|A}+q_{B|B})+q_{A|B}\right]. \quad [17d]$$

B.4. Change in $p_{AA}^{(i)}$**.** We proceed with the change in the frequency of each type of edge. Note that when a random player l is chosen to die, the edges between (i) l and its nearest neighbors and (ii) l's nearest neighbors and next-nearest neighbors have chance to update (see the description of local game transitions). We stress that the change in $p_{AA}^{(i)}$ is different from that in p_A. p_A does not change when neighboring A-players replace the focal A-player (the dead A-player) or neighboring B-players replace the focal B-player (the dead B-player). However, in the same case $p_{AA}^{(i)}$ likely changes since games in these edges may switch, which changes the edge type.

We first consider the case that a random B-player is chosen to die. We take the same neighborhood configuration as we do in Section B.1, i.e. $k_{A|B}^{(j)}, k_{B|B}^{(j)}$ for $j = 1, \ldots, n$. The change in $p_{AA}^{(i)}$ results from two parts: the switching of edges connecting the focal B-player and its nearest neighbors, and the switching of edges connecting the nearest neighbors and the next-nearest neighbors. Under the given neighborhood configuration, the change in $p_{AA}^{(i)}$ based on the former part is

$$\mathbb{P}\left(\Delta p_{AA}^{(i)} = \frac{2\left[\sum_{j=1}^{n} p_{ji}^{(1)} k_{A|B}^{(j)}\right]}{kN}\right) = \mathcal{B}\left(k_{A|B}^{(j)}, k_{B|B}^{(j)} | j = 1, \ldots, n\right) \mathbb{P}\left(A \to B\right). \qquad [18]$$

Eq. 18 describes the edge switching of $E_{BA}^{(j)} \to E_{AA}^{(i)}$, which occurs when (i) a neighboring A-player reproduces and its offspring replaces the dead B-player, i.e., $BA \to AA$; (ii) neighboring A-players who plays game j with the dead in the current time step then plays game i in the next time step, $(j) \to (i)$.

The change in $p_{AA}^{(i)}$ due to edges between the nearest and the next-nearest neighbors is

$$\begin{aligned}\mathbb{P}&\left(\Delta p_{AA}^{(i)} = \frac{2(k-1)\left[\sum_{j=1}^{n} p_{ji}^{(2)} q_{A|A}^{(j)} - \sum_{j=1}^{n} p_{ij}^{(2)} q_{A|A}^{(i)}\right] \sum_{j=1}^{n} k_{A|B}^{(j)}}{kN}\right) \\ &= \mathcal{B}\left(k_{A|B}^{(j)}, k_{B|B}^{(j)} | j = 1, \ldots, n\right) \left[\mathbb{P}\left(A \to B\right) + \mathbb{P}\left(B \to B\right)\right].\end{aligned} \qquad [19]$$

Eq. 19 indicates that regardless of which neighbor replaces the focal B-player, the change in $p_{AA}^{(i)}$ due to the edges between the nearest and next-nearest neighbors remains the same.

Next, we consider the case in which a random A-player is chosen to die. We take the same neighborhood configuration as we do in Section B.2, i.e., $k_{A|A}^{(j)}, k_{B|A}^{(j)}$ for $j = 1, \ldots, n$. The change in $p_{AA}^{(i)}$ due to edges between the focal A-player and its nearest neighbors is

$$\mathbb{P}\left(\Delta p_{AA}^{(i)} = \frac{2\left[\sum_{j=1}^{n} p_{ji}^{(2)} k_{A|A}^{(j)} - \sum_{j=1}^{n} p_{ij}^{(2)} k_{A|A}^{(i)}\right]}{kN}\right) = \mathcal{A}\left(k_{A|A}^{(j)}, k_{B|A}^{(j)} | j = 1, \ldots, n\right) \mathbb{P}\left(A \to A\right) \qquad [20]$$

and

$$\mathbb{P}\left(\Delta p_{AA}^{(i)} = \frac{-2k_{A|A}^{(i)}}{kN}\right) = \mathcal{A}\left(k_{A|A}^{(j)}, k_{B|A}^{(j)} | j = 1, \ldots, n\right) \mathbb{P}\left(B \to A\right). \qquad [21]$$

Eq. 20 (resp. Eq. 21) captures the case in which a neighboring A-player (resp. B-player) successfully occupies the vacant site.

The change in $p_{AA}^{(i)}$ due to edges between the nearest and next-nearest neighbors is

$$\begin{aligned}\mathbb{P}&\left(\Delta p_{AA}^{(i)} = \frac{2(k-1)\left[\sum_{j=1}^{n} p_{ji}^{(2)} q_{A|A}^{(j)} - \sum_{j=1}^{n} p_{ij}^{(2)} q_{A|A}^{(i)}\right] \sum_{j=1}^{n} k_{A|A}^{(j)}}{kN}\right) \\ &= \mathcal{A}\left(k_{A|A}^{(j)}, k_{B|A}^{(j)} | j = 1, \ldots, n\right) \left[\mathbb{P}\left(A \to A\right) + \mathbb{P}\left(B \to A\right)\right].\end{aligned} \qquad [22]$$

From Eqs. 18-22, the time derivative of $p_{AA}^{(i)}$ is given by

$$\begin{aligned}
\dot{p}_{AA}^{(i)} =& \sum_{\sum_{j=1}^{n}\left(k_{A|B}^{(j)}+k_{B|B}^{(j)}\right)=k} p_B \mathbb{P}\left(\Delta p_{AA}^{(i)} = \frac{2\sum_{j=1}^{n} p_{ji}^{(1)} k_{A|B}^{(j)}}{kN}\right) \frac{2\sum_{j=1}^{n} p_{ji}^{(1)} k_{A|B}^{(j)}}{kN} \\
&+ \sum_{\sum_{j=1}^{n}\left(k_{A|B}^{(j)}+k_{B|B}^{(j)}\right)=k} p_B \mathbb{P}\left(\Delta p_{AA}^{(i)} = \frac{2(k-1)\left[\sum_{j=1}^{n} p_{ji}^{(2)} q_{A|A}^{(j)} - \sum_{j=1}^{n} p_{ij}^{(2)} q_{A|A}^{(i)}\right]\sum_{j=1}^{n} k_{A|B}^{(j)}}{kN}\right) \\
&\qquad \frac{2(k-1)\left[\sum_{j=1}^{n} p_{ji}^{(2)} q_{A|A}^{(j)} - \sum_{j=1}^{n} p_{ij}^{(2)} q_{A|A}^{(i)}\right]\sum_{j=1}^{n} k_{A|B}^{(j)}}{kN} \\
&+ \sum_{\sum_{j=1}^{n}\left(k_{A|A}^{(j)}+k_{B|A}^{(j)}\right)=k} p_A \mathbb{P}\left(\Delta p_{AA}^{(i)} = \frac{2\left[\sum_{j=1}^{n} p_{ji}^{(2)} k_{A|A}^{(j)} - \sum_{j=1}^{n} p_{ij}^{(2)} k_{A|A}^{(i)}\right]}{kN}\right) \\
&\qquad \frac{2\left[\sum_{j=1}^{n} p_{ji}^{(2)} k_{A|A}^{(j)} - \sum_{j=1}^{n} p_{ij}^{(2)} k_{A|A}^{(i)}\right]}{kN} \\
&+ \sum_{\sum_{j=1}^{n}\left(k_{A|A}^{(j)}+k_{B|A}^{(j)}\right)=k} p_A \mathbb{P}\left(\Delta p_{AA}^{(i)} = \frac{-2k_{A|A}^{(i)}}{kN}\right) \frac{-2k_{A|A}^{(i)}}{kN} \\
&+ \sum_{\sum_{j=1}^{n}\left(k_{A|A}^{(j)}+k_{B|A}^{(j)}\right)=k} p_A \mathbb{P}\left(\Delta p_{AA}^{(i)} = \frac{2(k-1)\left[\sum_{j=1}^{n} p_{ji}^{(2)} q_{A|A}^{(j)} - \sum_{j=1}^{n} p_{ij}^{(2)} q_{A|A}^{(i)}\right]\sum_{j=1}^{n} k_{A|A}^{(j)}}{kN}\right) \\
&\qquad \frac{2(k-1)\left[\sum_{j=1}^{n} p_{ji}^{(2)} q_{A|A}^{(j)} - \sum_{j=1}^{n} p_{ij}^{(2)} q_{A|A}^{(i)}\right]\sum_{j=1}^{n} k_{A|A}^{(j)}}{kN} \\
=& \frac{2}{kN}\sum_{j=1}^{n}\left\{\left[k^2-(k-1)q_{B|A}\right]p_{ji}^{(2)} - k^2\delta_{ji}\right\}p_{AA}^{(j)} \\
&+ \frac{2}{kN}\sum_{j=1}^{n}\left[(k-1)q_{A|B}+1\right]p_{ji}^{(1)}p_{AB}^{(j)} + O(\delta),
\end{aligned} \qquad [23]$$

where $\delta_{ij} = 1$ if $i = j$ and $\delta_{ij} = 0$ otherwise.

B.5. Change in $p_{AB}^{(i)}$**.** When a B-player is selected to die and its neighbourhood configuration is the same as that in Section B.1, the change in $p_{AB}^{(i)}$ due to edges between the nearest and next-nearest neighbors is

$$\mathbb{P}\left(\Delta p_{AB}^{(i)} = \frac{-k_{A|B}^{(i)} + \sum_{j=1}^{n} p_{ji}^{(0)} k_{B|B}^{(j)}}{kN}\right) = \mathcal{B}\left(k_{A|B}^{(j)}, k_{B|B}^{(j)} | j = 1,\ldots,n\right)\mathbb{P}\left(A \to B\right) \qquad [24]$$

and

$$\mathbb{P}\left(\Delta p_{AB}^{(i)} = \frac{\sum_{j=1}^{n} p_{ji}^{(1)} k_{A|B}^{(j)} - \sum_{j=1}^{n} p_{ij}^{(1)} k_{A|B}^{(i)}}{kN}\right) = \mathcal{B}\left(k_{A|B}^{(j)}, k_{B|B}^{(j)} | j = 1,\ldots,n\right)\mathbb{P}\left(B \to B\right). \qquad [25]$$

Eq. 24 (resp. Eq. 25) captures the case when a neighboring A-player (resp. B-player) successfully occupies the vacant site.

The change in $p_{AB}^{(i)}$ due to edges between the nearest and next-nearest neighbors is

$$\begin{aligned}\mathbb{P}\Bigg(\Delta p_{AB}^{(i)} &= \frac{(k-1)\left[\sum_{j=1}^{n} p_{ji}^{(1)} q_{B|A}^{(j)} - \sum_{j=1}^{n} p_{ij}^{(1)} q_{B|A}^{(i)}\right] \sum_{j=1}^{n} k_{A|B}^{(j)}}{kN} \\ &\quad + \frac{(k-1)\left[\sum_{j=1}^{n} p_{ji}^{(1)} q_{A|B}^{(j)} - \sum_{j=1}^{n} p_{ij}^{(1)} q_{A|B}^{(i)}\right] \sum_{j=1}^{n} k_{B|B}^{(j)}}{kN}\Bigg) \\ &= \mathcal{B}\left(k_{A|B}^{(j)}, k_{B|B}^{(j)} | j=1,\ldots,n\right)\left[\mathbb{P}\left(A\to B\right) + \mathbb{P}\left(B \to B\right)\right].\end{aligned} \quad [26]$$

When an A-player is selected to die and its neighbourhood configuration is the same as that in Section B.2, the change in $p_{AB}^{(i)}$ due to edges between the nearest and next nearest neighbors is

$$\mathbb{P}\left(\Delta p_{AB}^{(i)} = \frac{\sum_{j=1}^{n} p_{ji}^{(1)} k_{B|A}^{(j)} - \sum_{j=1}^{n} p_{ij}^{(1)} k_{B|A}^{(i)}}{kN}\right) = \mathcal{A}\left(k_{A|A}^{(j)}, k_{B|A}^{(j)} | j=1,\ldots,n\right)\mathbb{P}\left(A \to A\right) \quad [27]$$

and

$$\mathbb{P}\left(\Delta p_{AB}^{(i)} = \frac{-k_{B|A}^{(i)} + \sum_{j=1}^{n} p_{ji}^{(2)} k_{A|A}^{(j)}}{kN}\right) = \mathcal{A}\left(k_{A|A}^{(j)}, k_{B|A}^{(j)} | j=1,\ldots,n\right)\mathbb{P}\left(B \to A\right). \quad [28]$$

Eq. 27 (resp. Eq. 28) captures the case when a neighboring A-player (resp. B-player) successfully occupies the vacant site.

The change in $p_{AB}^{(i)}$ due to edges between the nearest and next-nearest neighbors is

$$\begin{aligned}\mathbb{P}\Bigg(\Delta p_{AB}^{(i)} &= \frac{(k-1)\left[\sum_{j=1}^{n} p_{ji}^{(1)} q_{B|A}^{(j)} - \sum_{j=1}^{n} p_{ij}^{(1)} q_{B|A}^{(i)}\right] \sum_{j=1}^{n} k_{A|A}^{(j)}}{kN} \\ &\quad + \frac{(k-1)\left[\sum_{j=1}^{n} p_{ji}^{(1)} q_{A|B}^{(j)} - \sum_{j=1}^{n} p_{ij}^{(1)} q_{A|B}^{(i)}\right] \sum_{j=1}^{n} k_{B|A}^{(j)}}{kN}\Bigg) \\ &= \mathcal{A}\left(k_{A|A}^{(j)}, k_{B|A}^{(j)} | j=1,\ldots,n\right)\left[\mathbb{P}\left(A\to A\right) + \mathbb{P}\left(B \to A\right)\right].\end{aligned} \quad [29]$$

Analogously, we have

$$\begin{aligned}\dot{p}_{AB}^{(i)} =& \frac{1}{kN}\sum_{j=1}^{n}(k-1)q_{B|A}p_{ji}^{(2)}p_{AA}^{(j)} \\ &+ \frac{1}{kN}\sum_{j=1}^{n}\left[(k-1)(q_{A|A}+q_{B|B}+2k)p_{ji}^{(1)} - 2k^2\delta_{ji}\right]p_{AB}^{(j)} \\ &+ \frac{1}{kN}\sum_{j=1}^{n}(k-1)q_{A|B}p_{ji}^{(0)}p_{BB}^{(j)} + O(\delta).\end{aligned} \quad [30]$$

B.6. Change in $p_{BB}^{(i)}$***.*** When a B-player is selected to die and its neighbourhood configuration is the same as that in Section B.1, the change in $p_{AB}^{(i)}$ due to edges between the nearest and next-nearest neighbors is

$$\mathbb{P}\left(\Delta p_{BB}^{(i)} = \frac{-2k_{B|B}^{(i)}}{kN}\right) = \mathcal{B}\left(k_{A|B}^{(j)}, k_{B|B}^{(j)} | j=1,\ldots,n\right)\mathbb{P}\left(A \to B\right) \quad [31]$$

and

$$\mathbb{P}\left(\Delta p_{BB}^{(i)} = \frac{\sum_{j=1}^{n} p_{ji}^{(0)} k_{B|B}^{(j)} - \sum_{j=1}^{n} p_{ij}^{(0)} k_{B|B}^{(i)}}{kN}\right) = \mathcal{B}\left(k_{A|B}^{(j)}, k_{B|B}^{(j)} | j=1,\ldots,n\right)\mathbb{P}\left(B \to B\right). \quad [32]$$

Eq. 31 (resp. Eq. 32) captures the case when a neighboring A-player (resp. B-player) successfully occupies the vacant site.

The change in $p_{BB}^{(i)}$ due to edges between the nearest and next-nearest neighbors is

$$\mathbb{P}\left(\Delta p_{BB}^{(i)}=\frac{2(k-1)\left[\sum_{j=1}^{n}p_{ji}^{(0)}q_{B|B}^{(j)}-\sum_{j=1}^{n}p_{ij}^{(0)}q_{B|B}^{(i)}\right]\sum_{j=1}^{n}k_{B|B}^{(j)}}{kN}\right) \\ =\mathcal{B}\left(k_{A|B}^{(j)},k_{B|B}^{(j)}|j=1,\ldots,n\right)\left[\mathbb{P}\left(A\to B\right)+\mathbb{P}\left(B\to B\right)\right]. \quad [33]$$

When an A-player is selected to die and its neighbourhood configuration is the same as that in Section B.2, the change in $p_{BB}^{(i)}$ due to edges between the nearest and next nearest neighbors is

$$\mathbb{P}\left(\Delta p_{BB}^{(i)}=\frac{2\sum_{j=1}^{n}p_{ji}^{(1)}k_{B|A}^{(j)}}{kN}\right)=\mathcal{A}\left(k_{A|A}^{(j)},k_{B|A}^{(j)}|j=1,\ldots,n\right)\mathbb{P}\left(B\to A\right). \quad [34]$$

The change in $p_{BB}^{(i)}$ due to edges between the nearest and next nearest neighbors is

$$\mathbb{P}\left(\Delta p_{AA}^{(i)}=\frac{2(k-1)\left[\sum_{j=1}^{n}p_{ji}^{(0)}q_{B|B}^{(j)}-\sum_{j=1}^{n}p_{ij}^{(0)}q_{B|B}^{(i)}\right]\sum_{j=1}^{n}k_{B|A}^{(j)}}{kN}\right) \\ =\mathcal{A}\left(k_{A|A}^{(j)},k_{B|A}^{(j)}|j=1,\ldots,n\right)\left[\mathbb{P}\left(A\to A\right)+\mathbb{P}\left(B\to A\right)\right]. \quad [35]$$

The derivative of $p_{BB}^{(i)}$ is

$$\dot{p}_{BB}^{(i)}=\frac{2}{kN}\sum_{j=1}^{n}\left[(k-1)q_{B|A}+1\right]p_{ji}^{(1)}p_{AB}^{(j)}+\frac{2}{kN}\sum_{j=1}^{n}\left\{\left[k^2-(k-1)q_{A|B}\right]p_{ji}^{(0)}-k^2\delta_{ji}\right\}p_{BB}^{(j)}+O\left(\delta\right). \quad [36]$$

B.7. Different time scales. From Eq. 23, we have

$$\dot{p}_{AA}=\sum_{i=1}^{n}\dot{p}_{AA}^{(i)}=\frac{2p_A}{kN(1-p_A)}(q_{A|A}-1)\left[(k-1)q_{A|A}-(k-2)p_A-1\right]+O\left(\delta\right) \quad [37]$$

and

$$\dot{q}_{A|A}=\frac{d}{dt}\left(\frac{p_{AA}}{p_A}\right)=\frac{2}{kN(1-p_A)}(q_{A|A}-1)\left[(k-1)q_{A|A}-(k-2)p_A-1\right]+O\left(\delta\right). \quad [38]$$

When the intensity of selection is weak ($\delta\ll 1$), $q_{A|A}$ reaches its equilibrium much faster than p_A (see Eqs. 16,38). Thus, the dynamical system converges quickly onto the slow manifold with $\dot{q}_{A|A}=0$, so we have

$$q_{A|A}=\frac{k-2}{k-1}p_A+\frac{1}{k-1}. \quad [39]$$

From Eqs. 3a-3f and 39, we find that for all $X,Y\in\{A,B\}$, p_{XY} and $q_{X|Y}$ are a function of p_A.

We define a function $\mathbf{A}\left(\mathbf{R}^{(s)}\right)$ mapping a set of $(n-1)\times(n-1)$ matrix $\mathbf{R}^{(s)}$ to a $3(n-1)\times 3(n-1)$ matrix, given by

$$\mathbf{A}\left(\mathbf{R}^{(s)}\right)=\begin{bmatrix}2(k^2-\alpha)\mathbf{R}^{(2)} & 2(\beta+1)\mathbf{R}^{(1)} & \mathbf{0}\\ \alpha\mathbf{R}^{(2)} & (2k^2-k)\mathbf{R}^{(1)} & \beta\mathbf{R}^{(0)}\\ \mathbf{0} & 2(\alpha+1)\mathbf{R}^{(1)} & 2(k^2-\beta)\mathbf{R}^{(0)}\end{bmatrix}, \quad [40]$$

where $\alpha=(k-2)(1-p_A)$ and $\beta=(k-2)p_A$. Then we use $\mathbf{P}^{(s)}$ in Eq. 2 to define two $(n-1)\times(n-1)$ matrices as follow

$$\bar{\mathbf{P}}^{(s)}=\begin{bmatrix}p_{11}^{(s)}-p_{n1}^{(s)} & \cdots & p_{(n-1)1}^{(s)}-p_{n1}^{(s)}\\ \vdots & \ddots & \vdots\\ p_{1(n-1)}^{(s)}-p_{n(n-1)}^{(s)} & \cdots & p_{(n-1)(n-1)}^{(s)}-p_{n(n-1)}^{(s)}\end{bmatrix},\quad \tilde{\mathbf{P}}^{(s)}=\begin{bmatrix}p_{n1}^{(s)} & \cdots & 0\\ \vdots & \ddots & \vdots\\ 0 & \cdots & p_{n(n-1)}^{(s)}\end{bmatrix}, \quad [41]$$

where $s \in \{0,1,2\}$. Let $\mathbf{b}$ denote a column vector with $3(n-1)$ entries: the first $n-1$ entries are p_{AA}; the next $n-1$ entries are p_{AB}; the last $n-1$ entries are p_{BB}. Let $\mathbf{v}$ denote a column vector $\left(p_{AA}^{(1)},\ldots,p_{AA}^{(n-1)},p_{AB}^{(1)},\ldots,p_{AB}^{(n-1)},p_{BB}^{(1)},\ldots,p_{BB}^{(n-1)}\right)^T$. Combining Eq. 39 and $p_{XY}^{(n)} = p_{XY} - \sum_{i=1}^{n-1} p_{XY}^{(i)}$, we can reduce the system of Eqs. 23,30,36 to

$$\dot{\mathbf{v}} = \frac{1}{kN}\left[\mathbf{A}\left(\bar{\mathbf{P}}^{(s)}\right) - 2k^2\mathbf{I}\right]\mathbf{v} + \frac{1}{kN}\mathbf{A}\left(\tilde{\mathbf{P}}^{(s)}\right)\mathbf{b} \equiv \bar{\mathbf{A}}\mathbf{v} + \bar{\mathbf{b}}. \qquad [42]$$

For a linear system described by Eq. 42, its equilibrium points can be obtained by solving the equation $\left[\mathbf{A}\left(\bar{\mathbf{P}}^{(s)}\right) - 2k^2\mathbf{I}\right]\mathbf{v} + \mathbf{A}\left(\tilde{\mathbf{P}}^{(s)}\right)\mathbf{b} = \mathbf{0}$. If for $0 < p_A < 1$, all eigenvalues of $\bar{\mathbf{A}}$ are negative numbers or complex numbers with negative real parts, the system is asymptotically stable and has a single equilibrium point given by (6)

$$\mathbf{v} = -\left[\mathbf{A}\left(\bar{\mathbf{P}}^{(s)}\right) - 2k^2\mathbf{I}\right]^{-1}\mathbf{A}\left(\tilde{\mathbf{P}}^{(s)}\right)\mathbf{b}. \qquad [43]$$

Regardless of the initial state of $\left(p_{AA}^{(1)},\ldots,p_{AA}^{(n-1)},p_{AB}^{(1)},\ldots,p_{AB}^{(n-1)},p_{BB}^{(1)},\ldots,p_{BB}^{(n-1)}\right)^T$, the system ultimately approaches to the equilibrium point. In other words, the initial fractions of various games do not affect the evolutionary outcome. We state that none of $\bar{\mathbf{A}}$'s eigenvalues can be positive, since this leads to a few terms in $\mathbf{v}$ increasing above 1 or decreasing below 0 (6), which is unrealistic in the current system. But $\bar{\mathbf{A}}$ may have zero eigenvalues. In such cases, the system described by Eq. 42 has more than one equilibrium point. The initial state of $\left(p_{AA}^{(1)},\ldots,p_{AA}^{(n-1)},p_{AB}^{(1)},\ldots,p_{AB}^{(n-1)},p_{BB}^{(1)},\ldots,p_{BB}^{(n-1)}\right)^T$ determines the equilibrium point that the system approaches. That is, the initial fractions of various games influence the evolutionary outcome. In Section 2, we provide an approach to efficiently evaluate the dependence of the evolutionary outcome to the initial fractions of various games.

B.8. Diffusion approximation. For given game transition matrices and the initial fractions of various games, by solving Eq. 42, we obtain $p_{AA}^{(i)}$, $p_{AB}^{(i)}$, and $p_{BB}^{(i)}$ as functions of p_A. Substituting $p_{AA}^{(i)}$, $p_{AB}^{(i)}$, and $p_{BB}^{(i)}$ into Eqs. 17a-17d and combining with Eq. 39, we have

$$I_{R_i} = (k-2)(k+1)(1-p_A)v_i \quad (\equiv I_{R_i}(p_A)); \qquad [44a]$$

$$I_{S_i} = \left[-(k-2)(k+1)p_A + k^2 - k - 1\right]v_{n+i-1} \quad (\equiv I_{S_i}(p_A)); \qquad [44b]$$

$$I_{T_i} = -\left[(k-2)(k+1)p_A + 1\right]v_{n+i-1} \quad (\equiv I_{T_i}(p_A)); \qquad [44c]$$

$$I_{P_i} = -(k-2)(k+1)p_A v_{2n+i-2} \quad (\equiv I_{P_i}(p_A)) \qquad [44d]$$

for $1 \leqslant i \leqslant n-1$. We obtain I_{R_n}, I_{S_n}, I_{T_n} and I_{P_n} by separately replacing v_i in Eq. 44a with $\left(p_{AA} - \sum_{i=1}^{n-1} v_i\right)$, v_{n+i-1} in Eqs. 44b and 44c with $\left(p_{AB} - \sum_{i=1}^{n-1} v_{n+i-1}\right)$, and v_{2n+i-2} in Eq. 44d with $\left(p_{BB} - \sum_{i=1}^{n-1} v_{2n+i-2}\right)$.

We consider a one-dimensional diffusion process of the random variable p_A. Within a short time interval Δt, we have

$$\begin{aligned}\mathbb{E}\left[\Delta p_A\right] &= \frac{1}{N}\mathbb{P}\left(\Delta p_A = \frac{1}{N}\right) + \left(-\frac{1}{N}\right)\mathbb{P}\left(\Delta p_A = -\frac{1}{N}\right) \\ &= \delta\frac{1}{kN}\sum_{i=1}^{n}\left(I_{R_i}R_i + I_{S_i}S_i + I_{T_i}T_i + I_{P_i}P_i\right)\Delta t \equiv \bar{E}(p_A)\Delta t;\end{aligned} \qquad [45a]$$

$$\begin{aligned}\mathrm{Var}\left[\Delta p_A\right] &= \left(\frac{1}{N}\right)^2\mathbb{P}\left(\Delta p_A = \frac{1}{N}\right) + \left(-\frac{1}{N}\right)^2\mathbb{P}\left(\Delta p_A = -\frac{1}{N}\right) \\ &= \frac{2(k-2)}{N^2(k-1)}p_A(1-p_A)\Delta t \equiv \bar{V}(p_A)\Delta t.\end{aligned} \qquad [45b]$$

The fixation probability $\phi_A(x)$ of A-players with initial frequency $p_A(t=0) = x$, satisfies the following differential equation [see Eq. (5.2.186) in Ref (7) and detailed derivation]:

$$0 = \bar{E}(x)\frac{d\phi_A(x)}{dx} + \frac{\bar{V}(x)}{2}\frac{d^2\phi_A(x)}{dx^2}. \qquad [46]$$

The solution to Eq. 46 is [see Eq (5.2.189) in Ref (7)]

$$\phi_A(x) = \frac{\int_0^x G(y)dy}{\int_0^1 G(y)dy}, \qquad [47]$$

where

$$\begin{aligned} G(y) &= \exp\left(-\int \frac{2\bar{E}(y)}{\bar{V}(y)}dy\right) \\ &= \exp\left(-\int \delta\frac{N(k-1)}{k(k-2)}\sum_{i=1}^{n}\left(\frac{I_{R_i}(y)}{y(1-y)}R_i + \frac{I_{S_i}(y)}{y(1-y)}S_i + \frac{I_{T_i}(y)}{y(1-y)}T_i + \frac{I_{P_i}(y)}{y(1-y)}P_i\right)dy\right) \\ &= 1-\delta\frac{N(k-1)}{k(k-2)}\sum_{i=1}^{n}\int\left(R_i\frac{I_{R_i}(y)}{y(1-y)} + S_i\frac{I_{S_i}(y)}{y(1-y)} + T_i\frac{I_{T_i}(y)}{y(1-y)} + P_i\frac{I_{P_i}(y)}{y(1-y)}\right)dy + O\left(\delta^2\right). \end{aligned} \qquad [48]$$

In Eq. 48, the third equality holds when δ is sufficiently small.

B.9. Fixation probability. In a population of B-players, when a fraction x of B-players mutates to A-players, the fixation probability of these A-players is

$$\begin{aligned} \phi_A(x) = x+\delta\frac{N(k-1)}{k(k-2)}\sum_{i=1}^{n}\Bigg\{ & \\ & x\int_0^1\left[\int\left(R_i\frac{I_{R_i}(y)}{y(1-y)} + S_i\frac{I_{S_i}(y)}{y(1-y)} + T_i\frac{I_{T_i}(y)}{y(1-y)} + P_i\frac{I_{P_i}(y)}{y(1-y)}\right)dy\right]dy \\ & -\int_0^x\left[\int\left(R_i\frac{I_{R_i}(y)}{y(1-y)} + S_i\frac{I_{S_i}(y)}{y(1-y)} + T_i\frac{I_{T_i}(y)}{y(1-y)} + P_i\frac{I_{P_i}(y)}{y(1-y)}\right)dy\right]dy\Bigg\} + O\left(\delta^2\right). \end{aligned} \qquad [49]$$

The fixation probability of a fraction x of B-players is

$$\phi_B(x) = 1-\phi_A(1-x). \qquad [50]$$

Then the ratio of fixation probabilities is

$$\begin{aligned} \frac{\phi_A(x)}{\phi_B(x)} = 1+\delta\frac{N(k-1)}{k(k-2)x}\sum_{i=1}^{n}\Bigg\{ & \\ & \int_{1-x}^1\left[\int\left(R_i\frac{I_{R_i}(y)}{y(1-y)} + S_i\frac{I_{S_i}(y)}{y(1-y)} + T_i\frac{I_{T_i}(y)}{y(1-y)} + P_i\frac{I_{P_i}(y)}{y(1-y)}\right)dy\right]dy \\ & -\int_0^x\left[\int\left(R_i\frac{I_{R_i}(y)}{y(1-y)} + S_i\frac{I_{S_i}(y)}{y(1-y)} + T_i\frac{I_{T_i}(y)}{y(1-y)} + P_i\frac{I_{P_i}(y)}{y(1-y)}\right)dy\right]dy\Bigg\} + O\left(\delta^2\right). \end{aligned} \qquad [51]$$

For sufficiently small x, we have

$$\begin{aligned} \frac{\phi_A(x)}{\phi_B(x)} = 1+\delta\frac{N(k-1)}{k(k-2)x}\sum_{i=1}^{n}\Bigg\{ & \\ & x\left[\int\left(R_i\frac{I_{R_i}(y)}{y(1-y)} + S_i\frac{I_{S_i}(y)}{y(1-y)} + T_i\frac{I_{T_i}(y)}{y(1-y)} + P_i\frac{I_{P_i}(y)}{y(1-y)}\right)dy\right]_{y=1} \\ & -x\left[\int\left(R_i\frac{I_{R_i}(y)}{y(1-y)} + S_i\frac{I_{S_i}(y)}{y(1-y)} + T_i\frac{I_{T_i}(y)}{y(1-y)} + P_i\frac{I_{P_i}(y)}{y(1-y)}\right)dy\right]_{y=0}\Bigg\} + O\left(\delta^2\right) \\ = 1+\delta\frac{N(k-1)}{k(k-2)}\sum_{i=1}^{n}\Bigg[& R_i\int_0^1\frac{I_{R_i}(y)}{y(1-y)}dy + S_i\int_0^1\frac{I_{S_i}(y)}{y(1-y)}dy \\ & + T_i\int_0^1\frac{I_{T_i}(y)}{y(1-y)}dy + P_i\int_0^1\frac{I_{P_i}(y)}{y(1-y)}dy\Bigg] + O\left(\delta^2\right). \end{aligned} \qquad [52]$$

Overall, for a sufficiently large population and $x = 1/N$, the condition of A-players being favored over B-players ($\rho_A > \rho_B$) is

$$\sum_{i=1}^{n}\left[R_i\int_0^1 \frac{I_{R_i}(y)}{y(1-y)}dy + S_i\int_0^1 \frac{I_{S_i}(y)}{y(1-y)}dy + T_i\int_0^1 \frac{I_{T_i}(y)}{y(1-y)}dy + P_i\int_0^1 \frac{I_{P_i}(y)}{y(1-y)}dy\right] > 0. \qquad [53]$$

Eq. 53 holds for not only death-birth updating, but also for imitation (see Section 1C for details) and pairwise-comparison (see Section 1D for details) updating. Note that for different updating rules, I_{R_i}, I_{S_i}, I_{T_i}, I_{P_i} differ.

B.10. The rule $b/c > k - k'$. We now turn to donation games. The payoff structure for game i is

$$\begin{array}{cc} & \begin{array}{cc} A & B \end{array} \\ \begin{array}{c} A \\ B \end{array} & \begin{pmatrix} b_i - c & -c \\ b_i & 0 \end{pmatrix}. \end{array} \qquad [54]$$

Substituting payoff structures into Eq. 53 and using Eqs. 17a-17d, we obtain the condition for $\rho_A > \rho_B$ under death-birth updating, given by

$$\sum_{i=1}^{n} \alpha_i b_i + \alpha_c c > 0, \qquad [55]$$

where

$$\alpha_i = \int_0^1 \frac{I_{R_i}(y) + I_{T_i}(y)}{y(1-y)}dy, \qquad \alpha_c = -\frac{k^2(k-2)}{k-1}. \qquad [56]$$

Furthermore, we have

$$\sum_{i=1}^{n} \alpha_i = \frac{k(k-2)}{k-1}\left(= -\frac{\alpha_c}{k}\right). \qquad [57]$$

Using $\alpha_1 = -\alpha_c/k - \sum_{i=2}^{n}\alpha_i$ and denoting $(b_1 - b_i)/c$ by $\Delta b_{1i}/c$, we can rewrite Eq. 55 as

$$\frac{b_1}{c} > k - \sum_{i=2}^{n} \xi_i \frac{\Delta b_{1i}}{c}, \qquad [58]$$

where

$$\xi_i = -\frac{k-1}{k(k-2)}\int_0^1 \frac{I_{R_i}(y) + I_{T_i}(y)}{y(1-y)}dy. \qquad [59]$$

Inserting Eqs. 44a and 44c into Eq. 59, we get the formula of ξ_i for death-birth updating. Letting $k' = \sum_{i=2}^{n}\xi_i \Delta b_{1i}/c$ and $b = b_1$, we obtain the rule $b/c > k - k'$.

C. Imitation updating. In each time step, a random player i is selected to evaluate its strategy. This player retains its own strategy or imitates a neighbor's strategy with probability proportional to fitness. Analyzing the evolutionary process as we do under death-birth updating, we have

$$\dot{p}_A = \delta\frac{k}{(k+1)^2 N}\sum_{i=1}^{n}\left(I_{R_i}R_i + I_{S_i}S_i + I_{T_i}T_i + I_{P_i}P_i\right) + O\left(\delta^2\right), \qquad [60]$$

where

$$I_{R_i} = p_{AA}^{(i)}(k-1)q_{B|A}\left[(k-1)(q_{A|A} + q_{B|B}) + 3\right]; \qquad [61a]$$

$$I_{S_i} = p_{AB}^{(i)}\left\{(k-1)q_{B|A}\left[(k-1)(q_{A|A} + q_{B|B}) + 2\right] + (k-1)q_{B|B} + 2\right\}; \qquad [61b]$$

$$I_{T_i} = -p_{AB}^{(i)}\left\{(k-1)q_{A|B}\left[(k-1)(q_{A|A} + q_{B|B}) + 2\right] + (k-1)q_{A|A} + 2\right\}; \qquad [61c]$$

$$I_{P_i} = -p_{BB}^{(i)}(k-1)q_{A|B}\left[(k-1)(q_{A|A} + q_{B|B}) + 3\right]. \qquad [61d]$$

We redefine the function $\mathbf{A}\left(\mathbf{R}^{(s)}\right)$ to be

$$\mathbf{A}\left(\mathbf{R}^{(s)}\right) = \begin{bmatrix} 2(k^2+k-\alpha)\mathbf{R}^{(2)} & 2(\beta+1)\mathbf{R}^{(1)} & \mathbf{0} \\ \alpha\mathbf{R}^{(2)} & (2k^2+k)\mathbf{R}^{(1)} & \beta\mathbf{R}^{(0)} \\ \mathbf{0} & 2(\alpha+1)\mathbf{R}^{(1)} & 2(k^2+k-\beta)\mathbf{R}^{(0)} \end{bmatrix}. \qquad [62]$$

Then the system under imitation updating can be reduced to

$$\dot{\mathbf{v}} = \frac{1}{kN}\left[\mathbf{A}\left(\bar{\mathbf{P}}^{(s)}\right) - 2k(k+1)\mathbf{I}\right]\mathbf{v} + \frac{1}{kN}\mathbf{A}\left(\tilde{\mathbf{P}}^{(s)}\right)\mathbf{b}. \quad [63]$$

All other variables such as $\alpha, \beta, \bar{\mathbf{P}}^{(s)}, \tilde{\mathbf{P}}^{(s)}, \mathbf{b}, \mathbf{v}$ follow those defined for death-birth updating.

For donation games described by Eq. 54, we have the condition for $\rho_A > \rho_B$,

$$\frac{b_1}{c} > k + 2 - \sum_{i=2}^{n} \xi_i \frac{\Delta b_{1i}}{c}, \quad [64]$$

where

$$\xi_i = -\frac{(k-1)}{k(k-2)} \int_0^1 \frac{I_{R_i}(y) + I_{T_i}(y)}{y(1-y)} dy. \quad [65]$$

Solving Eq. 63 and inserting I_{R_i}, I_{T_i} in Eq. 65, we get the expression for ξ_i.

D. Pairwise-comparison updating. In each generation, a random player i is selected to evaluate its strategy. This player randomly selects a neighbor j and compares payoffs. Player i then adopts j's strategy with probability

$$\frac{1}{1 + e^{-\delta(\pi_j - \pi_i)}}, \quad [66]$$

where π_i and π_j denote the payoffs of i and j, respectively. Otherwise, player i retains its strategy.

Analogously, we have

$$\dot{p}_A = \delta \frac{1}{2N} \sum_{i=1}^{n} \left(I_{R_i} R_i + I_{S_i} S_i + I_{T_i} T_i + I_{P_i} P_i\right) + O\left(\delta^2\right), \quad [67]$$

where

$$I_{R_i} = p_{AA}^{(i)}(k-1) q_{B|A}; \quad [68a]$$

$$I_{S_i} = p_{AB}^{(i)} \left[(k-1) q_{B|A} + 1\right]; \quad [68b]$$

$$I_{T_i} = -p_{AB}^{(i)} \left[(k-1) q_{A|B} + 1\right]; \quad [68c]$$

$$I_{P_i} = -p_{BB}^{(i)}(k-1) p_{A|B}. \quad [68d]$$

We redefine the function $\mathbf{A}\left(\mathbf{R}^{(s)}\right)$ to be

$$\mathbf{A}\left(\mathbf{R}^{(s)}\right) = \begin{bmatrix} 2(4k-2-\alpha)\mathbf{R}^{(2)} & 2(\beta+1)\mathbf{R}^{(1)} & \mathbf{0} \\ \alpha\mathbf{R}^{(2)} & (7k-4)\mathbf{R}^{(1)} & \beta\mathbf{R}^{(0)} \\ \mathbf{0} & 2(\alpha+1)\mathbf{R}^{(1)} & 2(4k-2-\beta)\mathbf{R}^{(0)} \end{bmatrix}. \quad [69]$$

Then the system under pairwise-comparison updating can be reduced to

$$\dot{\mathbf{v}} = \frac{1}{kN}\left[\mathbf{A}\left(\bar{\mathbf{P}}^{(s)}\right) - (8k-4)\mathbf{I}\right]\mathbf{v} + \frac{1}{kN}\mathbf{A}\left(\tilde{\mathbf{P}}^{(s)}\right)\mathbf{b}. \quad [70]$$

All other variables such as $\alpha, \beta, \bar{\mathbf{P}}^{(s)}, \tilde{\mathbf{P}}^{(s)}, \mathbf{b}, \mathbf{v}$ follow those defined for death-birth updating.

For donation games described by Eq. 54, we have the condition for $\rho_A > \rho_B$,

$$\sum_{i=2}^{n} \xi_i \frac{\Delta b_{1i}}{c} > 1, \quad [71]$$

where

$$\xi_i = -\frac{(k-1)}{k(k-2)} \int_0^1 \frac{I_{R_i}(y) + I_{T_i}(y)}{y(1-y)} dy. \quad [72]$$

By solving Eq. 70 and inserting I_{R_i}, I_{T_i} in Eq. 72, we get the expression for ξ_i.

2. Approach to evaluate the sensitivity of evolutionary dynamics to the initial condition

Here, we consider the initial condition, which refers to the initial fractions of various games played in the population. By calculating the eigenvalues of matrix $\left[\mathbf{A}\left(\bar{\mathbf{P}}^{(s)}\right)-2k^2\mathbf{I}\right]/(kN)$ in Eq. 42 and evaluating the sign of all eigenvalues, we can tell whether or not under a given game transition pattern the evolutionary outcome is sensitive to the initial condition under death-birth updating for local game transitions. Analogously, we can study the matrix $\left[\mathbf{A}\left(\bar{\mathbf{P}}^{(s)}\right)-2k(k+1)\mathbf{I}\right]/(kN)$ in Eq. 63 under imitation updating and the matrix $\left[\mathbf{A}\left(\bar{\mathbf{P}}^{(s)}\right)-(8k-4)\mathbf{I}\right]/(kN)$ in Eq. 70 under pairwise-comparison updating.

In this section, we provide an alternative approach to determine the dependence of the evolutionary outcome on the initial conditions. Based on the game transition matrix $\mathbf{P}^{(2)}$, $\mathbf{P}^{(1)}$, and $\mathbf{P}^{(0)}$ in Eq. 2, we define a Markov chain with a state space $\mathbf{E}=\{1,2,\ldots,3n\}$. The probability transition matrix for this Markov chain is given by

$$\mathbf{M}=\begin{bmatrix}\mathbf{P}^{(2)}/2 & \mathbf{P}^{(2)}/2 & \mathbf{0}\\ \mathbf{P}^{(1)}/3 & \mathbf{P}^{(1)}/3 & \mathbf{P}^{(1)}/3\\ \mathbf{0} & \mathbf{P}^{(0)}/2 & \mathbf{P}^{(0)}/2\end{bmatrix}. \quad [73]$$

The entry in the ith row and the jth column of $\mathbf{M}$ is the transition probability from state i to state j. If the defined random process has only one closed communicating class, the evolutionary outcome is independent of the initial condition, regardless of the update rule. However, if it has more than one such class, the evolutionary outcome is sensitive to the initial condition.

For a random process defined by a state space $\mathbf{E}$ and a probability transition matrix $\mathbf{M}$, we can examine its communicating class structure as follows: letting $\bar{\mathbf{M}}=\sum_{i=1}^{3n}\mathbf{M}^i$, the above random process has only one closed communicating class if and only if in $\bar{\mathbf{M}}$ there exists at least some i $(1\leqslant i\leqslant 3n)$ such that all entries in the ith column are positive. The random process has more than one closed communicating classes if and only if in $\bar{\mathbf{M}}$ for every i $(1\leqslant i\leqslant 3n)$ there exists at least one entry of 0 in the ith column.

The sign of each entry in $\bar{\mathbf{M}}$, taking $\bar{M}_{ij}$ (the entry in the ith row and the jth column in $\bar{\mathbf{M}}$) for example, actually indicates the transition possibility (not probability) from state i to state j within $3n$-step transitions (less than or equal to $3n$ steps). $\bar{M}_{ij}>0$ means that the system can transition from state i to j in at most $3n$ steps. For $\bar{M}_{ij}=0$, the transition is unlikely to happen within $3n$ steps, which indicates that the system entering into state i can never transition to state j. If there exists some j $(1\leqslant j\leqslant 3n)$ such that all entries in the jth column of $\bar{\mathbf{M}}$ are positive, any state can transition to state j and thus state j lies in a closed communicating class. In such a situation, if there is another closed communicating class, any state lying in the second class is unlikely to transition to state j, which leads to a contradiction. Therefore, a column of positive entries suggests a single closed communicating class. Similarly, if there is only one closed communicating class, any state can transition to one state of the closed communicating class. Thus, there must exist a column of positive entries. Analogously, we can prove that the absence of a column of positive entries implies the existence of more than one closed communicating class. We provide examples with two states for a better understanding of this approach. The game transition matrices are

$$\mathbf{P}^{(2)}=\begin{bmatrix}1 & 0\\ 0 & 1\end{bmatrix},\quad \mathbf{P}^{(1)}=\begin{bmatrix}1 & 0\\ 0 & 1\end{bmatrix},\quad \mathbf{P}^{(0)}=\begin{bmatrix}1 & 0\\ 0 & 1\end{bmatrix}. \quad [74]$$

We have

$$\mathbf{M}=\begin{bmatrix}1/2 & 0 & 1/2 & 0 & 0 & 0\\ 0 & 1/2 & 0 & 1/2 & 0 & 0\\ 1/3 & 0 & 1/3 & 0 & 1/3 & 0\\ 0 & 1/3 & 0 & 1/3 & 0 & 1/3\\ 0 & 0 & 1/2 & 0 & 1/2 & 0\\ 0 & 0 & 0 & 1/2 & 0 & 1/2\end{bmatrix}, \quad [75]$$

which gives

$$\bar{\mathbf{M}}=\begin{bmatrix}631/290 & 0 & 129/49 & 0 & 56/47 & 0\\ 0 & 631/290 & 0 & 129/49 & 0 & 56/47\\ 86/49 & 0 & 122/49 & 0 & 86/49 & 0\\ 0 & 86/49 & 0 & 122/49 & 0 & 86/49\\ 56/47 & 0 & 129/49 & 0 & 631/290 & 0\\ 0 & 56/47 & 0 & 122/49 & 0 & 631/290\end{bmatrix}. \quad [76]$$

There exist entries of 0 in every column of $\bar{\mathbf{M}}$. Thus, there is more than one closed communicating class, and the initial fractions of various games affect the evolutionary outcomes. As a consistency check, we calculate the eigenvalues of $\left[\mathbf{A}\left(\bar{\mathbf{P}}^{(s)}\right)-2k^2\mathbf{I}\right]/(kN)$ in Eq. 42, which are given by $\lambda_1=0$, $\lambda_2=-(k-2)/(Nk)$, and $\lambda_3=-(2k-2)/(Nk)$. The eigenvalue $\lambda_1=0$ confirms the sensitivity of the evolutionary outcome to the initial conditions.

Actually, Eq. 74 intuitively shows that the game remains fixed throughout the evolutionary process. According to prior studies about edge-dependent games, the evolution proceeds "as if" all interactions are governed by an "effective" game (8, 9). This "effective" game corresponds to the averaged of games played in all interactions, which suggests that its payoff structure depends on the fractions of various games. Thus the evolutionary outcome is sensitive to the initial condition, in line with the analysis based on the above approach. In Section 4C, we illustrate how to calculate ξ_i for such a system.

Then, we present an example with game transition matrices

$$\mathbf{P}^{(2)}=\begin{bmatrix}1&0\\1&0\end{bmatrix},\quad \mathbf{P}^{(1)}=\begin{bmatrix}0&1\\0&1\end{bmatrix},\quad \mathbf{P}^{(0)}=\begin{bmatrix}0&1\\0&1\end{bmatrix}. \qquad [77]$$

Actually, this case corresponds to the game transition pattern used in Fig. 1 in the main text: both taking strategy A leads to game 1 and other strategy profiles lead to game 2. We have

$$\mathbf{M}=\begin{bmatrix}1/2&0&1/2&0&0&0\\1/2&0&1/2&0&0&0\\0&1/3&0&1/3&0&1/3\\0&1/3&0&1/3&0&1/3\\0&0&0&1/2&0&1/2\\0&0&0&1/2&0&1/2\end{bmatrix}, \qquad [78]$$

which gives

$$\bar{\mathbf{M}}=\begin{bmatrix}49/34&36/49&49/34&56/47&0&56/47\\49/34&36/49&49/34&56/47&0&56/47\\36/49&50/49&36/49&86/49&0&86/49\\36/49&50/49&36/49&86/49&0&86/49\\37/81&36/49&37/81&631/290&0&631/290\\37/81&36/49&37/81&631/290&0&631/290\end{bmatrix}. \qquad [79]$$

Except for entries in the fifth column, all other entries in $\bar{\mathbf{M}}$ are positive. There is only one closed communicating class. The evolutionary outcome therefore is insensitive to the initial condition. As a consistency check, we calculate the eigenvalues of $\left[\mathbf{A}\left(\bar{\mathbf{P}}^{(s)}\right)-2k^2\mathbf{I}\right]/(kN)$ in Eq. 42, which are given by $\lambda_1=-2k/N$, $\lambda_2=-2k/N$, and $\lambda_3=-2k/N$. The system has a unique equilibrium point and the evolutionary outcome is independent of the initial condition. In Section 4C, we illustrate how to calculate ξ_i for this system.

We now briefly explain why the closed communicating class of this random process can predict the sensitivity of the evolutionary outcome to the initial condition. The main idea is whether or not the initially-assigned game between two connected players constrains the game they play in the long-term evolutionary process. For example, the transition pattern we illustrate in Eq. 74 describes that if two individuals initially play game 1, regardless of their strategic actions, they will play game 1 throughout the process. Obviously, the initial game decides the game they play later. Therefore, the initial condition affects the evolutionary outcome.

We rename the states in $\mathbf{E}$ as $\left\{E_{AA}^{(1)},\ldots,E_{AA}^{(n)},E_{AB}^{(1)},\ldots,E_{AB}^{(n)},E_{BB}^{(1)},\ldots,E_{BB}^{(n)}\right\}$, where the i_{th} entry corresponds to the original state i. In the following, we show that $\mathbf{M}$ actually captures the state transition of an edge throughout the process. As defined in Section 1, the state of an edge is given by $E_{XY}^{(i)}$, where $X,Y\in\{A,B\}$ and $i\in\{1,2,\ldots,n\}$. X and Y are strategies of the two connected players and i is the game they play. The transition of an edge state can arise from two parts: the change in players' strategies and the change in the game they play. The illustrated below is the transition of an edge in one time step:

$$\begin{array}{ccccc}E_{AA}^{(i)} & \underset{p_{ji}^{(1)}}{\overset{p_{ij}^{(2)}}{\rightleftharpoons}} & E_{AB}^{(j)} & \underset{p_{lj}^{(0)}}{\overset{p_{jl}^{(1)}}{\rightleftharpoons}} & E_{BB}^{(l)}\\ p_{ii_1}^{(2)}\downharpoonleft\upharpoonright p_{i_1i}^{(2)} & & p_{jj_1}^{(1)}\downharpoonleft\upharpoonright p_{j_1j}^{(1)} & & p_{ll_1}^{(0)}\downharpoonleft\upharpoonright p_{l_1l}^{(0)}\\ E_{AA}^{(i_1)} & & E_{AB}^{(j_1)} & & E_{BB}^{(l_1)}\end{array} \qquad [80]$$

Let AA denote both players taking A-strategies, AB denote one player taking $A-$strategy and the other taking B-strategy, and BB denote both players taking $B-$strategies. Note that in the current model, in each generation, only a player has the opportunity to update its strategy. Thus, the strategy transition follows (i) AA can remain in AA or transition to AB but can not transition to BB; (ii) AB can remain in AB or transition to AA or BB; (iii) BB can remain in BB or transition to AB but can not transition to AA. That is, for any i and l, $E_{AA}^{(i)}$ and $E_{BB}^{(l)}$ are unlikely to transition to each other, corresponding to the two null matrices in $\mathbf{M}$ (see $\mathbf{0}$ in Eq. 73). The transition of the game is governed by $\mathbf{P}^{(2)}$, $\mathbf{P}^{(1)}$, and $\mathbf{P}^{(0)}$. For example, $\mathbf{P}^{(2)}$ determines whether or not an edge of $E_{AA}^{(i)}$ can transition to $E_{AA}^{(i_1)}$ or $E_{AB}^{(j)}$ in a time step ($i_1, j \in \{1, 2, \ldots, n\}$), corresponding to those terms including $\mathbf{P}^{(2)}$ in $\mathbf{M}$. Note that the realistic evolutionary process is much more complicated and it is impossible to obtain the exact transition probability of an edge from one state to another, but the matrix $\mathbf{M}$ can describe the possibility that an edge of $E_{X_1Y_1}^{(i)}$ transitions to that of $E_{X_2Y_2}^{(j)}$ in a time step. The zero entries in $\mathbf{M}$ indicate the transition can never happen and the nonzero entries suggest the transition is can happen.

When the random process has only closed communicating class, let $\mathbf{c}$ denote the set of all states lying in this class. Eqs. 16,23,30,36 show that for a sufficiently small δ, the fractions of various edges change much faster than the fractions of the two strategies. That is, although there is a frequent transition between A-players and B-players, namely an A-player transitioning to a B-player or a B-player transitioning to an A-player, the fraction of A-players varies at a relatively low rate. In the evolutionary process, an edge transitions among various states as the strategies adopted by the connected individuals and the game they play change. The state transition possibility of an edge is described by $\mathbf{M}$. Eventually, the state of this edge enters into the closed communicating class $\mathbf{c}$ and can never escape from $\mathbf{c}$, regardless of its initial state. Thus, if the random process defined above has only one closed communicating class, the evolutionary outcome is independent of the initial condition.

When the random process has m (>1) closed communicating classes, we denote them by $\mathbf{c}_1, \mathbf{c}_2, \ldots, \mathbf{c}_m$. If it is possible for an edge of $E_{X_1Y_1}^{(i)}$ to transition to $E_{X_2Y_2}^{(j)}$ within one update step, we denote it by $E_{X_1Y_1}^{(i)} \to E_{X_2Y_2}^{(j)}$. We apply two propositions below:

(i) for $E_{X_1Y_1}^{(i_1)} \in \mathbf{c}_{j_1}$ and $E_{X_2Y_2}^{(i_2)} \in \mathbf{c}_{j_2}$, $i_1 \neq i_2$ for any $j_1 \neq j_2$ regardless of X_1Y_1 and X_2Y_2;

(ii) in every closed communicating class $\mathbf{c}_j$, there exists some i satisfying $E_{AA}^{(i)} \in \mathbf{c}_j$, $E_{AB}^{(i)} \in \mathbf{c}_j$ and $E_{BB}^{(i)} \in \mathbf{c}_j$. For different closed communicating classes, i can be different.

About proposition (i), for $E_{AA}^{(i)} \in \mathbf{c}_j$, since $E_{AA}^{(i)}$ is a recurrent state, there exists l making either $E_{AA}^{(l)} \to E_{AA}^{(i)}$ or $E_{AB}^{(l)} \to E_{AA}^{(i)}$. Note that l can be i. $E_{BB}^{(l)} \to E_{AA}^{(i)}$ is impossible since in each generation just one player has the change to update its strategy. $E_{AA}^{(l)} \to E_{AA}^{(i)}$ means $E_{AA}^{(l)} \to E_{AB}^{(i)}$ due to the strategy transition, leading to $E_{AB}^{(i)} \in \mathbf{c}_j$. $E_{AB}^{(l)} \to E_{AA}^{(i)}$ means $E_{AB}^{(l)} \to E_{AB}^{(i)}$ due to the game transition, leading to $E_{AB}^{(i)} \in \mathbf{c}_j$. Thus, $E_{AA}^{(i)} \in \mathbf{c}_j$ always gives $E_{AB}^{(i)} \in \mathbf{c}_j$. Similarly, $E_{BB}^{(i)} \in \mathbf{c}_j$ gives $E_{AB}^{(i)} \in \mathbf{c}_j$. That is, $E_{X_1Y_1}^{(i_1)} \in \mathbf{c}_{j_1}$ means $E_{AB}^{(i_1)} \in \mathbf{c}_{j_1}$ and $E_{X_2Y_2}^{(i_2)} \in \mathbf{c}_{j_2}$ means $E_{AB}^{(i_2)} \in \mathbf{c}_{j_2}$. For $j_1 \neq j_2$, $i_1 \neq i_2$. Otherwise, an edge state lies in two different closed communicating classes, which leads to a contradiction.

Based on the proof of proposition (i), every closed communicating class includes at lease one state with the form of $E_{AB}^{(i)}$. Note that i in different closed communicating classes is different. Let $E_{AB}^{(i)} \in \mathbf{c}_j$. Due to the game transition, there must exist l with $E_{AB}^{(i)} \to E_{AB}^{(l)}$ and $E_{AB}^{(l)} \in \mathbf{c}_j$. In addition, due to the strategy transition, we have $E_{AB}^{(i)} \to E_{AA}^{(l)}$ and $E_{AB}^{(i)} \to E_{BB}^{(l)}$, which gives $E_{AA}^{(l)} \in \mathbf{c}_j$ and $E_{BB}^{(l)} \in \mathbf{c}_j$. Overall, $\mathbf{c}_j$ includes $E_{AA}^{(l)}$, $E_{AB}^{(l)}$ and $E_{BB}^{(l)}$.

Proposition (i) stresses that when an edge transitions into a state that lies in a closed communicating class like $\mathbf{c}_{j_1}$, the games to be played by the two connected players are limited by $\mathbf{c}_{j_1}$. If the edge transitions into a state in another closed communicating class like $\mathbf{c}_{j_2}$, the two connected players can only play games limited by $\mathbf{c}_{j_2}$. In particular, the games limited by $\mathbf{c}_{j_1}$ and those by $\mathbf{c}_{j_2}$ are completely different. The initial condition affects which closed communicating class an edge will transition into. A representative example is that for $E_{AA}^{(i_1)} \in \mathbf{c}_{j_1}$, $E_{AB}^{(i_1)} \in \mathbf{c}_{j_1}$, $E_{BB}^{(i_1)} \in \mathbf{c}_{j_1}$ and $E_{AA}^{(i_2)} \in \mathbf{c}_{j_2}$, $E_{AB}^{(i_2)} \in \mathbf{c}_{j_2}$, $E_{BB}^{(i_2)} \in \mathbf{c}_{j_2}$, when all players play game i_1 initially, the games to be played are limited by $\mathbf{c}_{j_1}$ throughout the process. However, if initially all players play game i_2, $\mathbf{c}_{j_2}$ constrains the games to be played throughout the process.

We examine the above approach with 10^8 numerical examples. In every example, we generate three 4×4 random matrices in which all entries are nonnegative. We normalize these matrices to make the sum of entries in each row 1. The three matrices are assigned to $\mathbf{P}^{(2)}$, $\mathbf{P}^{(1)}$, and $\mathbf{P}^{(0)}$. On the one hand, based on $\mathbf{P}^{(2)}$, $\mathbf{P}^{(1)}$, and $\mathbf{P}^{(0)}$, we calculate the eigenvalues of $\left[\mathbf{A}\left(\bar{\mathbf{P}}^{(s)}\right) - 2k^2\mathbf{I}\right]/(kN)$ in Eq. 42 and record whether or not there are zero eigenvalues. On the other, based on $\mathbf{P}^{(2)}$, $\mathbf{P}^{(1)}$, and $\mathbf{P}^{(0)}$, we calculate $\bar{\mathbf{M}}$ and record whether or not there is some i such that all entries

in the ith column of $\bar{\mathbf{M}}$ are positive. In all examples, whenever there is zero entry in each column of $\bar{\mathbf{M}}$, there are zero eigenvalues. As long as there exists some i such that all entries in the ith column of $\bar{\mathbf{M}}$ are positive, there is no zero eigenvalue. Thus, our approach predicts well the sensitivity of the evolutionary outcomes to the initial condition. Furthermore, for $\mathbf{P}^{(2)}$, $\mathbf{P}^{(1)}$, and $\mathbf{P}^{(0)}$ under which the evolutionary outcome is sensitive to the initial condition, a slight perturbation or noise to the game transition pattern (to $\mathbf{P}^{(2)}$, $\mathbf{P}^{(1)}$, and $\mathbf{P}^{(0)}$) can turn this system into one insensitive to the initial condition. A simple way to achieve this is adding to each entry in $\mathbf{P}^{(2)}$, $\mathbf{P}^{(1)}$, and $\mathbf{P}^{(0)}$ an arbitrary small number δ_1, δ_2, and δ_3, respectively, where δ_1, δ_2, and δ_3 are not necessary identical.

3. Evolutionary dynamics with global game transitions

A. Global game transitions. In this section, we study evolutionary dynamics with global game transitions. In each time step, games in all interactions have chances to update. We proceed with the mathematical analysis as we do in Section 1. We take the same variables and notations (Eqs. 2-3f). The change in p_A follows Eqs. 16-17d. We then investigate the change in the frequency of each type of edge. Assuming that a random B-player is selected to die, the change in $p_{AA}^{(i)}$ arises from two parts: the switching of edges connecting the focal (dead) B-player and its nearest neighbors, the switching of all other edges. Under the neighborhood configuration given in Section B.1, Eq. 18 shows the change in $p_{AA}^{(i)}$ due to the former part. The change in $p_{AA}^{(i)}$ due to the latter is

$$\begin{aligned}&\mathbb{P}\left(\Delta p_{AA}^{(i)}=\frac{\sum_{j=1}^{n}Nkp_{AA}^{(j)}p_{ji}^{(2)}-\sum_{j=1}^{n}Nkp_{AA}^{(i)}p_{ij}^{(2)}}{kN}\right)\\&=\mathcal{B}\left(k_{A|B}^{(j)},k_{B|B}^{(j)}|j=1,\ldots,n\right)\left[\mathbb{P}\left(A\to B\right)+\mathbb{P}\left(B\to B\right)\right].\end{aligned} \tag{81}$$

Suppose that a random A-player is selected to die. Under the neighborhood configuration given in Section B.2, Eqs. 20 and 21 capture the change in $p_{AA}^{(i)}$ due to edges between the focal A-player and its nearest neighbors. The change in $p_{AA}^{(i)}$ due to the switching of other edges is

$$\begin{aligned}&\mathbb{P}\left(\Delta p_{AA}^{(i)}=\frac{\sum_{j=1}^{n}\left(Nkp_{AA}^{(j)}-2k_{A|A}^{(j)}\right)p_{ji}^{(2)}-\sum_{j=1}^{n}\left(Nkp_{AA}^{(i)}-2k_{A|A}^{(i)}\right)p_{ij}^{(2)}}{kN}\right)\\&=\mathcal{A}\left(k_{A|A}^{(j)},k_{B|A}^{(j)}|j=1,\ldots,n\right)\left[\mathbb{P}\left(A\to A\right)+\mathbb{P}\left(B\to A\right)\right].\end{aligned} \tag{82}$$

From Eqs. 18,20,21,81,82, we obtain the time derivative of $p_{AA}^{(i)}$, given by

$$\dot{p}_{AA}^{(i)}=\sum_{j=1}^{n}p_{AA}^{(j)}p_{ji}^{(2)}-p_{AA}^{(i)}-\frac{2}{kN}\sum_{j=1}^{n}(k-1)q_{B|A}p_{ji}^{(2)}p_{AA}^{(j)}+\frac{2}{kN}\sum_{j=1}^{n}\left[(k-1)q_{A|B}+1\right]p_{ji}^{(1)}p_{AB}^{(j)}+O\left(\delta\right). \tag{83}$$

Analogously, we obtain the time derivatives of $p_{AB}^{(i)}$ and $p_{BB}^{(i)}$, given by

$$\begin{aligned}\dot{p}_{AB}^{(i)}=&\sum_{j=1}^{n}p_{AB}^{(j)}p_{ji}^{(1)}-p_{AB}^{(i)}\\&+\frac{1}{kN}\sum_{j=1}^{n}(k-1)q_{B|A}p_{ji}^{(2)}p_{AA}^{(j)}\\&+\frac{1}{kN}\sum_{j=1}^{n}\left[(k-1)\left(q_{A|A}+q_{B|B}\right)-2k\right]p_{ji}^{(1)}p_{AB}^{(j)}\\&+\frac{1}{kN}\sum_{j=1}^{n}(k-1)q_{A|B}p_{ji}^{(0)}p_{BB}^{(j)}+O\left(\delta\right)\end{aligned} \tag{84}$$

and

$$\dot{p}_{BB}^{(i)}=\sum_{j=1}^{n}p_{BB}^{(j)}p_{ji}^{(0)}-p_{BB}^{(i)}-\frac{2}{kN}\sum_{j=1}^{n}(k-1)q_{A|B}p_{ji}^{(0)}p_{BB}^{(j)}+\frac{2}{kN}\sum_{j=1}^{n}\left[(k-1)q_{B|A}+1\right]p_{ji}^{(1)}p_{AB}^{(j)}+O\left(\delta\right). \tag{85}$$

Analogous to Eqs. 37 and 38, a further analysis to Eq. 83 gives Eq. 39. We redefine the function $\mathbf{A}\left(\mathbf{R}^{(s)}\right)$ to be

$$\mathbf{A}\left(\mathbf{R}^{(s)}\right) = \begin{bmatrix} (1-2\alpha/\mu)\mathbf{R}^{(2)} & 2(1+\beta)/\mu\mathbf{R}^{(1)} & \mathbf{0} \\ \alpha/\mu\mathbf{R}^{(2)} & (1-k/\mu)\mathbf{R}^{(1)} & \beta/\mu\mathbf{R}^{(0)} \\ \mathbf{0} & 2(1+\alpha)/\mu\mathbf{R}^{(1)} & (1-2\beta/\mu)\mathbf{R}^{(0)} \end{bmatrix}, \tag{86}$$

where $\mu = kN$. α and β follow those defined in Eq. 40. We can reduce the system of Eqs. 83-85 to

$$\dot{\mathbf{v}} = \left[\mathbf{A}\left(\bar{\mathbf{P}}^{(s)}\right) - \mathbf{I}\right]\mathbf{v} + \mathbf{A}\left(\tilde{\mathbf{P}}^{(s)}\right)\mathbf{b}, \tag{87}$$

where $\bar{\mathbf{P}}^{(s)}, \tilde{\mathbf{P}}^{(s)}, \mathbf{b}, \mathbf{v}$ are defined in Eqs. 41 and 42. Similarly, solving Eq. 87, substituting the solutions into Eqs. 44a-44d, and inserting $I_{R_i}, I_{S_i}, I_{T_i}, I_{P_i}$ into Eq. 53, we obtain the condition of one strategy being favored over the other under general two-strategy games. In particular, when all games are donation games, Eq. 58 predicts when A is favored over B. ξ_i can be obtained by inserting I_{R_i} and I_{T_i} into Eq. 59. The approach proposed in Section 2 still determines whether or not the evolutionary dynamics is sensitive to the initial condition under global game transitions.

B. A class of game transition patterns. We provide an alternative approach to study a class of game transition patterns (denoted by Ω): for every $s \in \{0,1,2\}$, in a Markov chain $\{M_t^{(s)}, t = 0,1,2,3,\dots\}$ with state space $\{1,2,\dots,n\}$ and state transition matrix $\mathbf{P}^{(s)}$, there is only one recurrent equivalence class (and the states therein are aperiodic). Introducing $\mathbf{u}^{(s)} = \left(u_1^{(s)},\dots,u_n^{(s)}\right)$, the solution to $\mathbf{u}^{(s)} = \mathbf{u}^{(s)}\mathbf{P}^{(s)}$ with $\sum_{j=1}^n u_i^{(s)} = 1$ is the limiting distribution of this Markov chain.

In each time step, only one among N players has the chance to modify its strategy whereas all games are likely to update. The evolutionary rate of the game in an interaction (or in an edge) is $N/2$ times as large as that of interactants' strategies. Therefore, for sufficiently large population size N, the fractions of various games reach a stationary distribution much faster than the fractions of various strategies. For games between two A-players, the stationary distribution is $\mathbf{u}^{(2)}$. Thus, in the interaction of two A-players, the expected payoff for each A-player is $\sum_{i=1}^n u_i^{(2)} R_i$. Analogously, the stationary distribution for games between an A-player and a B-player is $\mathbf{u}^{(1)}$. The expected payoff for A-player is $\sum_{i=1}^n u_i^{(1)} S_i$ and that of B-player is $\sum_{i=1}^n u_i^{(1)} T_i$. For games between two B-players, the stationary distribution is $\mathbf{u}^{(0)}$. The expected payoff for each defector is $\sum_{i=1}^n u_i^{(0)} P_i$. The game transition creates a situation "as if" all players play an "effective" game, with payoff structure

$$\begin{array}{c} \\ A \\ B \end{array}\!\!\begin{array}{c} \begin{array}{cc} \quad A \quad\quad & \quad\quad B \end{array} \\ \begin{pmatrix} \sum_{i=1}^n u_i^{(2)} R_i & \sum_{i=1}^n u_i^{(1)} S_i \\ \sum_{i=1}^n u_i^{(1)} T_i & \sum_{i=1}^n u_i^{(0)} P_i \end{pmatrix} \end{array} \equiv \begin{array}{c} \\ A \\ B \end{array}\!\!\begin{array}{c} \begin{array}{cc} A & B \end{array} \\ \begin{pmatrix} \bar{R} & \bar{S} \\ \bar{T} & \bar{P} \end{pmatrix} \end{array}, \tag{88}$$

which holds for death-birth, imitation, pairwise-comparison, and birth-death updating.

Under death-birth updating, in Eq. 53, replacing R_i, S_i, T_i and P_i with $\bar{R}$, $\bar{S}$, $\bar{T}$ and $\bar{P}$, then inserting Eqs. 44a-44d, we reduce Eq. 53 to

$$(k+1)\sum_{i=1}^n u_i^{(2)} R_i + (k-1)\sum_{i=1}^n u_i^{(1)} S_i > (k-1)\sum_{i=1}^n u_i^{(1)} T_i + (k+1)\sum_{i=1}^n u_i^{(0)} P_i. \tag{89}$$

For donation games with $R_i = b_i - c$, $S_i = -c$, $T_i = b_i$ and $P_i = 0$, Eq. 89 is further reduced to Eq. 58 with

$$\xi_i = -\frac{(k+1)u_i^{(2)} - (k-1)u_i^{(1)}}{2}. \tag{90}$$

Similarly, under imitation updating, we can reduce Eq. 53 to

$$(k+3)\sum_{i=1}^n u_i^{(2)} R_i + (k+1)\sum_{i=1}^n u_i^{(1)} S_i > (k+1)\sum_{i=1}^n u_i^{(1)} T_i + (k+3)\sum_{i=1}^n u_i^{(0)} P_i. \tag{91}$$

A further analysis of donation games leads to Eq. 64 with

$$\xi_i = -\frac{(k+3)u_i^{(2)} - (k+1)u_i^{(1)}}{2}. \tag{92}$$

Under both pairwise-comparison and birth-death updating, A-players are favored over B-players if and only if

$$\sum_{i=1}^{n} u_i^{(2)} R_i + \sum_{i=1}^{n} u_i^{(1)} S_i > \sum_{i=1}^{n} u_i^{(1)} T_i + \sum_{i=1}^{n} u_i^{(0)} P_i. \quad [93]$$

A simplification for donation games gives Eq. 71 with

$$\xi_i = -\frac{u_i^{(2)} - u_i^{(1)}}{2}. \quad [94]$$

In this section, we solve $\mathbf{u}^{(s)} = \mathbf{u}^{(s)}\mathbf{P}^{(s)}$ and use the limiting distribution $\mathbf{u}^{(s)}$ to approximate the evolutionary process. Eqs. 83-85 actually imply this idea. For weak selection ($\delta \ll 1$), $p_{AA}^{(i)}$, $p_{AB}^{(i)}$, $p_{BB}^{(i)}$ reach the equilibrium point much faster than p_A (see Eqs. 16 and 83-85). The dynamical system thus converges quickly onto the slow manifold with $\dot{p}_{AA}^{(i)} = 0$, $\dot{p}_{AB}^{(i)} = 0$, $\dot{p}_{BB}^{(i)} = 0$. In the righthand of Eq. 83, $1/N$ occurs in the third and fourth terms. For a sufficiently large population size N ($N \gg 1$), the existence of $1/N$ may make the two terms negligible relative to the first and second terms. This inspires the idea of using $\sum_{j=1}^{n} p_{AA}^{(j)} p_{ji}^{(2)} - p_{AA}^{(i)} = 0$ to approximate $\dot{p}_{AA}^{(i)} = 0$. Replacing $p_{AA}^{(j)}$ with $u_j^{(2)}$, we have $\mathbf{u}^{(2)}\mathbf{P}^{(2)} = \mathbf{u}^{(2)}$. The analogous analysis to Eqs. 83 and 84 gives $\mathbf{u}^{(1)}\mathbf{P}^{(1)} = \mathbf{u}^{(1)}$ and $\mathbf{u}^{(0)}\mathbf{P}^{(0)} = \mathbf{u}^{(0)}$.

C. Game transitions in a fraction of interactions. With global game transitions, games in all interactions have chances to update in each time step. With local game transitions, games in a fraction of interactions have chances to update in each time step. Note that with local game transitions, the interactions allowing for game transitions are spatially correlated—only games in interactions involved with the deceased individual's neighbors are likely to update. In this section, we assume that in each time step a fraction p ($0 < p < 1$) of games are randomly selected to update. In other words, in each interaction, the game has chance to update with probability p and has no chance to update with probability $1 - p$. Equivalently, in each interaction, with probability p the game transitions based on the probability matrix $\mathbf{P}^{(s)}$, and with probability $1 - p$ the game transitions to itself. Such a situation corresponds to game transitions based on a new probability matrix $\hat{\mathbf{P}}^{(s)}$,

$$\hat{\mathbf{P}}^{(s)} = p\mathbf{P}^{(s)} + (1-p)\mathbf{I}.$$

Note that the solution to $\mathbf{u}^{(s)} = \mathbf{u}^{(s)}\hat{\mathbf{P}}^{(s)}$ is the same as $\mathbf{u}^{(s)} = \mathbf{u}^{(s)}\mathbf{P}^{(s)}$. The setting of a fraction of games being transitioned thus leads to the same results as global transition does.

D. Stochastic strategies. Up to this point, all players are assumed to be using pure strategies, namely a player cooperating (taking A) unconditionally or a player defecting (adopting B) unconditionally in each time step. In the following, we further investigate the case where players take stochastic strategies, i.e. choosing to cooperate with a probability and to defect otherwise. Let s_p and s_q denote two stochastic strategies. Players taking s_p choose to cooperate with probability p and defect with probability $1 - p$. If taking s_q, players choose cooperation with probability q and defection with probability $1 - q$. For two connected players, before one of them has a chance to update its strategy (i.e. s_p or s_q), their actions (i.e. cooperation or defection) and games they played update many times. Therefore, in a sufficiently large population, the fractions of various interaction scenarios (consisting of two actions and the game they play) reach a stationary distribution much faster than the fractions of various strategies.

We study the competition between s_p and s_q. By taking $p = 1$ (pure cooperators) and $q = 0$ (pure defectors), this model can recover the case of pure strategies. In the following, we calculate the stationary distribution of interaction scenarios between players taking s_p and players taking s_q. Let $u_{i,r1,r2}^{(pq)}(t)$ denote the probability that in time t a player with s_p chooses action r_1, a player with s_q chooses action r_2, and they play game i, where $i \in \{1, \dots, n\}$ and $r_1, r_2 \in \{0, 1\}$ (0 represents defection and 1 means cooperation). Then, we have

$$u_{j,1,1}^{(pq)}(t+1) = \sum_{i=1}^{n} \left[u_{i,1,1}^{(pq)}(t) p_{ij}^{(2)} + u_{i,1,0}^{(pq)}(t) p_{ij}^{(1)} + u_{i,0,1}^{(pq)}(t) p_{ij}^{(1)} + u_{i,0,0}^{(pq)}(t) p_{ij}^{(0)} \right] pq; \quad [95a]$$

$$u_{j,1,0}^{(pq)}(t+1) = \sum_{i=1}^{n} \left[u_{i,1,1}^{(pq)}(t) p_{ij}^{(2)} + u_{i,1,0}^{(pq)}(t) p_{ij}^{(1)} + u_{i,0,1}^{(pq)}(t) p_{ij}^{(1)} + u_{i,0,0}^{(pq)}(t) p_{ij}^{(0)} \right] p(1-q); \quad [95b]$$

$$u_{j,0,1}^{(pq)}(t+1) = \sum_{i=1}^{n} \left[u_{i,1,1}^{(pq)}(t) p_{ij}^{(2)} + u_{i,1,0}^{(pq)}(t) p_{ij}^{(1)} + u_{i,0,1}^{(pq)}(t) p_{ij}^{(1)} + u_{i,0,0}^{(pq)}(t) p_{ij}^{(0)} \right] (1-p)q; \quad [95c]$$

$$u_{j,0,0}^{(pq)}(t+1)=\sum_{i=1}^{n}\left[u_{i,1,1}^{(pq)}(t)p_{ij}^{(2)}+u_{i,1,0}^{(pq)}(t)p_{ij}^{(1)}+u_{i,0,1}^{(pq)}(t)p_{ij}^{(1)}+u_{i,0,0}^{(pq)}(t)p_{ij}^{(0)}\right](1-p)(1-q). \quad [95d]$$

For the game transition pattern Ω, there exists a stationary distribution and we denote it by $\mathbf{u}^{(pq)}=(u_{1,1,1}^{(pq)},\ldots,u_{n,1,1}^{(pq)},u_{1,1,0}^{(pq)},\ldots,u_{n,1,0}^{(pq)},u_{1,0,1}^{(pq)},\ldots,u_{n,0,1}^{(pq)},u_{1,0,0}^{(pq)},\ldots,u_{n,0,0}^{(pq)})$, where $u_{i,r_1,r_2}^{(pq)}$ indicates the stationary fraction of interactions in which two players play game i and the one with strategy s_p chooses action r_1 and the other with strategy s_q chooses action r_2. We rewrite Eqs. 95a-95d as

$$\mathbf{u}^{(pq)}=\mathbf{u}^{(pq)}\begin{bmatrix} pq\mathbf{P}^{(2)} & p(1-q)\mathbf{P}^{(2)} & (1-p)q\mathbf{P}^{(2)} & (1-p)(1-q)\mathbf{P}^{(2)} \\ pq\mathbf{P}^{(1)} & p(1-q)\mathbf{P}^{(1)} & (1-p)q\mathbf{P}^{(1)} & (1-p)(1-q)\mathbf{P}^{(1)} \\ pq\mathbf{P}^{(1)} & p(1-q)\mathbf{P}^{(1)} & (1-p)q\mathbf{P}^{(1)} & (1-p)(1-q)\mathbf{P}^{(1)} \\ pq\mathbf{P}^{(0)} & p(1-q)\mathbf{P}^{(0)} & (1-p)q\mathbf{P}^{(0)} & (1-p)(1-q)\mathbf{P}^{(0)} \end{bmatrix}. \quad [96]$$

We can get the stationary distribution $\mathbf{u}^{(pq)}$ by the left eigenvector with $\sum_{r_1=0}^{1}\sum_{r_2=0}^{1}\sum_{i=1}^{n}u_{i,r_1,r_2}^{pq}=1$. Actually, letting $\bar{\mathbf{u}}^{(pq)}=(u_1^{(pq)},\ldots,u_n^{(pq)})$ and solving

$$\bar{\mathbf{u}}^{(pq)}=\bar{\mathbf{u}}^{(pq)}\left[pq\mathbf{P}^{(2)}+(p+q-2pq)\mathbf{P}^{(1)}+(1-p)(1-q)\mathbf{P}^{(0)}\right] \quad [97]$$

with $\sum_{i=1}^{n}u_i^{pq}=1$, we have $u_{i,1,1}^{(pq)}=pqu_i^{(pq)}$, $u_{i,1,0}^{(pq)}=p(1-q)u_i^{(pq)}$, $u_{i,0,1}^{(pq)}=(1-p)qu_i^{(pq)}$, and $u_{i,0,0}^{(pq)}=(1-p)(1-q)u_i^{(pq)}$. In the interaction between a player with strategy s_p and a player with strategy s_q, the former's expected payoff is

$$f_{pq}=\sum_{i=1}^{n}u_i^{(pq)}\left[pqR_i+p(1-q)S_i+(1-p)qT_i+(1-p)(1-q)P_i\right]. \quad [98]$$

Under death-birth updating, the condition for strategy s_p to be favored over s_q (i.e. $\rho_{s_p}>\rho_{s_q}$) is

$$(k+1)f_{pp}+(k-1)f_{pq}>(k-1)f_{qp}+(k+1)f_{qq}. \quad [99]$$

We say that a stochastic strategy is more cooperative if players with such a strategy choose cooperation with a larger probability. That is, for $p>q$, s_p is more cooperative than s_q. For donation games described by Eq. 54, Eq. 99 can be reduced to Eq. 58 with

$$\xi_i=\frac{-(k+1)pu_i^{(pp)}-(k-1)qu_i^{(pq)}+(k-1)pu_i^{(qp)}+(k+1)qu_i^{(qq)}}{2(p-q)}. \quad [100]$$

Similarly, under imitation updating in donation games, natural selection favors s_p over s_q if Eq. 64 holds, where

$$\xi_i=\frac{-(k+3)pu_i^{(pp)}-(k+1)qu_i^{(pq)}+(k+1)pu_i^{(qp)}+(k+3)qu_i^{(qq)}}{2(p-q)}. \quad [101]$$

For birth-death or pairwise-comparison updating in donation games, s_p is favored over s_q if Eq. 71 holds, where

$$\xi_i=\frac{-pu_i^{(pp)}-qu_i^{(pq)}+pu_i^{(qp)}+qu_i^{(qq)}}{2(p-q)}. \quad [102]$$

E. Intuition based on "sigma rule". Here we provide a few new insights into how game transitions affect the evolution of A-players. In the game between A-players and B-players governed by a single payoff matrix

$$\begin{array}{cc} & \begin{array}{cc} A & B \end{array} \\ \begin{array}{c} A \\ B \end{array} & \begin{pmatrix} R & S \\ T & P \end{pmatrix} \end{array}, \quad [103]$$

Tarnita et al have found that selection favors A-players over B-players if and only if

$$\sigma R+S>T+\sigma P, \quad [104]$$

which is termed as "sigma rule" (10). The coefficient σ captures how the spatial model and its associated update rule affect evolutionary dynamics, whereas is independent of the payoffs. For an infinite random regular graph under death-birth updating, $\sigma = (k+1)/(k-1)$.

When all interactions are governed by a fixed donation game with a donation cost c and benefit b_1, substituting $R = b_1 - c$, $S = -c$, $T = b_1$ and $P = 0$ into the sigma rule gives the condition of A-players being favored over B-players. Intriguingly, Eq. 58 can be phrased in the form of a sigma rule with $R = b_1 - c + \frac{2}{k+1}\sum_{i=2}^{n}\xi_i \Delta b_{1i}$, $S = -c$, $T = b_1$ and $P = 0$. With game transitions, evolution proceeds "as if" all interactions are governed by an effective game with payoff matrix

$$\begin{array}{c} \\ A \\ B \end{array} \begin{array}{c} \begin{array}{cc} A & B \end{array} \\ \begin{pmatrix} b_1 - c + \frac{2}{k+1}\sum_{i=2}^{n}\xi_i \Delta b_{1i} & -c \\ b_1 & 0 \end{pmatrix} \end{array}. \quad [105]$$

Compared with the fixed donation game, mutual cooperation brings each player an extra payoff $\frac{2}{k+1}\sum_{i=2}^{n}\xi_i \Delta b_{1i}$ in this effective game. This payoff depends on two factors: game transition patterns (described by ξ_i), variations in different games (described by Δb_{1i}).

For an infinite random regular graph under pairwise-comparison updating, $\sigma = 1$. Analogously, Eq. 71 can be phrased in the form of a sigma rule with $R = b_1 - c + 2\sum_{i=2}^{n}\xi_i \Delta b_{1i}$, $S = -c$, $T = b_1$ and $P = 0$. With game transitions, evolution proceeds "as if" all interactions are governed by an effective game with payoff structure

$$\begin{array}{c} \\ A \\ B \end{array} \begin{array}{c} \begin{array}{cc} A & B \end{array} \\ \begin{pmatrix} b_1 - c + 2\sum_{i=2}^{n}\xi_i \Delta b_{1i} & -c \\ b_1 & 0 \end{pmatrix} \end{array}. \quad [106]$$

4. Representative examples

In Section 1 and 3, we derive the general condition of one strategy to be favored over the other strategy, which requires to solve a set of equations. In this section, we study four representative interaction scenarios and provide explicit expressions.

A. Evolutionary dynamics with state-independent game transitions. If the game to be played in the next time step is independent of the game played in the past, the game transition is state-independent. That is, $p_{im}^{(s)} = p_{jm}^{(s)}$ for all i and j. The number of A-players determines the game to be played. Let $p_{im}^{(s)} = p_m^{(s)}$. For local game transitions, focusing on game transition patterns under which the evolutionary outcome is independent of the initial condition, we have

$$\begin{aligned} \mathbf{DB}: \xi_i &= \frac{\left(-6k^4 - 2k^3 - 3k^2 - 6k - 2\right)p_i^{(2)} + \left(6k^4 - 11k^3 + 3k^2 + 6k + 4\right)p_i^{(1)} + \left(k^3 - 2\right)p_i^{(0)}}{12k^3}; \\ \mathbf{IM}: \xi_i &= \frac{\left(-6k^4 - 20k^3 - 13k^2 - 14k - 6\right)p_i^{(2)} + \left(6k^4 + 7k^3 - k^2 + 10k + 12\right)p_i^{(1)} + \left(k^3 + 2k^2 + 4k - 6\right)p_i^{(0)}}{12k^2(k+1)}; \\ \mathbf{PC}: \xi_i &= \frac{\left(-20k^2 + 8k - 2\right)p_i^{(2)} + \left(19k^2 - 10k + 4\right)p_i^{(1)} + \left(k^2 + 2k - 2\right)p_i^{(0)}}{24k(2k-1)}. \end{aligned} \quad [107]$$

For global game transitions, focusing on the game transition pattern Ω introduced in Section 3, we have

$$\begin{aligned} \mathbf{DB}: \quad \xi_i &= -\frac{(k+1)p_i^{(2)} - (k-1)p_i^{(1)}}{2}; \\ \mathbf{IM}: \quad \xi_i &= -\frac{(k+3)p_i^{(2)} - (k+1)p_i^{(1)}}{2}; \\ \mathbf{PC/DB}: \quad \xi_i &= -\frac{p_i^{(2)} - p_i^{(1)}}{2}. \end{aligned} \quad [108]$$

In particular, if $p_m^{(s)} = 1/n$ for all m and s, the game transition is fully stochastic. In the next time step, any game occurs with the equal probability. For both local and global game transitions, weak selection favors A over B if

$$\begin{aligned} &\textbf{DB}: \quad (k+1)\sum_{i=1}^{n} R_i + (k-1)\sum_{i=1}^{n} S_i - (k-1)\sum_{i=1}^{n} T_i - (k+1)\sum_{i=1}^{n} P_i > 0; \\ &\textbf{IM}: \quad (k+3)\sum_{i=1}^{n} R_i + (k+1)\sum_{i=1}^{n} S_i - (k+1)\sum_{i=1}^{n} T_i - (k+3)\sum_{i=1}^{n} P_i > 0; \\ &\textbf{PC}: \quad \sum_{i=1}^{n} R_i + \sum_{i=1}^{n} S_i - \sum_{i=1}^{n} T_i - \sum_{i=1}^{n} P_i > 0. \end{aligned} \qquad [109]$$

Let $\bar{R} = \left(\sum_{i=1}^{n} R_i\right)/n$, $\bar{S} = \left(\sum_{i=1}^{n} S_i\right)/n$, $\bar{T} = \left(\sum_{i=1}^{n} T_i\right)/n$, $\bar{P} = \left(\sum_{i=1}^{n} P_i\right)/n$. We find that the evolutionary process with stochastic and diverse games can be approximated by that of a static and unified game.

B. Evolutionary dynamics with strategy-independent game transitions. If the game to be played in the next time step is independent of players' strategic actions in the past, the game transition is strategy-independent. That is, $\mathbf{P}^{(2)} = \mathbf{P}^{(1)} = \mathbf{P}^{(0)}$. Let $\mathbf{P}^{(2)} = \mathbf{P}^{(1)} = \mathbf{P}^{(0)} = \mathbf{P}$. Here, we consider the game transition pattern Ω introduced in Section 3.

For global game transitions, we introduce a vector $\mathbf{u} = (u_1, u_2, \ldots, u_n)$, which satisfies $\mathbf{u} = \mathbf{uP}$. Then, we have

$$\begin{aligned} &\textbf{DB}: \xi_i = -u_i; \\ &\textbf{IM}: \xi_i = -u_i; \\ &\textbf{PC/BD}: \quad \xi_i = 0. \end{aligned} \qquad [110]$$

Note that in pairwise-comparison or birth-death updating, $\xi_i = 0$ means that cooperation can never evolve regardless of the benefit provided by a cooperative behavior in the donation game. In other words, if the game is independent of strategic actions, game transitions cannot promote cooperation.

C. Evolutionary dynamics with game transitions between two states ($n = 2$). Given the theoretical significance of two states, we provide a systematic investigation of game transitions between two donation games. According to Eq. 58, under death-birth updating, the general rule for cooperation to be favored over defection is

$$\frac{b_1}{c} > k - \xi \frac{\Delta b}{c}. \qquad [111]$$

For local game transitions, focusing on game transition patterns under which the evolutionary outcome is insensitive to the initial condition, we have

$$\xi = \int_0^1 \frac{\beta_3 y^3 + \beta_2 y^2 + \beta_1 y + \beta_0}{\alpha_2 y^2 + \alpha_1 y + \alpha_0} dy, \qquad [112]$$

where

$$\begin{aligned}
\alpha_2 &= -k(P_0-1)\left[2(P_1-1)(P_2-1)k^4+(P_1+2P_2-3P_1P_2)k^3+(5P_1P_2-4P_2)k^2-3P_1P_2k+2P_1P_2\right];\\
\alpha_1 &= k(k-2)\left[2(P_2-P_0)(P_1-1)k^2+2(P_0-P_1)P_2k+P_0P_1-4P_0P_2+3P_1P_2\right];\\
\alpha_0 &= k(k-2)^2(P_0P_1-2P_0P_2+P_1P_2);\\
\beta_3 &= (k+1)(k-2)^3\left[p_{21}^{(2)}(P_1-P_0)+p_{21}^{(1)}(P_0-P_2)+p_{21}^{(0)}(P_2-P_1)\right];\\
\beta_2 &= -p_{21}^{(2)}(k-2)^2\left[(P_0-2P_1+1)k^3+(1-P_0)k^2+(3P_0-2P_1)k+4P_0-4P_1\right];\\
&\quad -p_{21}^{(1)}(k-2)^2\left[(2P_2-2)k^3+(2P_0-2)k^2+(P_2-3P_0)k-4P_0+4P_2\right]\\
&\quad -p_{21}^{(0)}(k-2)^2\left[(1-P_2)k^3+(P_2-2P_1+1)k^2+(2P_1-P_2)k+4P_1-4P_2\right]+\alpha_0;\\
\beta_1 &= p_{21}^{(2)}(k-2)\big[-2(P_0-1)(P_1-1)k^5+P_1(P_0-1)k^4+(P_0+4P_1-2P_0P_1-3)k^3\\
&\qquad -2(P_0-1)(P_1-2)k^2+P_0(P_1-2)k-3P_0+5P_1-2P_0P_1\big]\\
&\quad +p_{21}^{(1)}(k-2)\big[(2P_0-2)(P_2-1)k^5-(P_0-1)(P_2-1)k^4+(2P_2-3)(P_0-1)k^3\\
&\qquad +(2P_0P_2-4P_2-6P_0+6)k^2+(2P_0+3P_2-P_0P_2)k+3P_0-5P_2+2P_0P_2\big]\\
&\quad +p_{21}^{(0)}(k-2)\left[(1-P_2)k^4+(P_2-P_1)k^3+(4P_1-2)k^2-3P_2k-5P_1+5P_2\right]+\alpha_1;\\
\beta_0 &= -p_{21}^{(2)}(1-P_0)\left[(2-2P_1)k^5+P_1k^4+(2-2P_1)k^3+(3-2P_1)k^2+P_1k-2P_1\right]\\
&\quad +p_{21}^{(1)}(1-P_0)\left[-2P_2k^4+(3P_2+1)k^3+(4-5P_2)k^2+3P_2k-2P_2\right]\\
&\quad -p_{21}^{(0)}\left[(P_1+P_2-1)k^3+(1-2P_2-2P_1)k^2+(3P_2-P_1)k+2P_1-2P_2\right]+\alpha_2.
\end{aligned} \qquad [113]$$

In the above equations, $P_2=p_{11}^{(2)}-p_{21}^{(2)}$, $P_1=p_{11}^{(1)}-p_{21}^{(1)}$, and $P_0=p_{11}^{(0)}-p_{21}^{(0)}$.

For global game transitions, focusing on the game transition pattern Ω introduced in Section 3, we have

$$\xi=\frac{(k-1)p_{12}^{(1)}p_{21}^{(2)}-(k+1)p_{21}^{(1)}p_{12}^{(2)}-2p_{12}^{(1)}p_{12}^{(2)}}{2\left(p_{12}^{(1)}+p_{21}^{(1)}\right)\left(p_{12}^{(2)}+p_{21}^{(2)}\right)}. \qquad [114]$$

We proceed with a specific game transition pattern involving two states: mutual cooperation leads to game 1 and all other action profiles lead to game 2. Replacing A with cooperation (C) and B with defection (D), the game transition matrix is Eq. 77. In Section 2, we have shown that the evolutionary process is insensitive to the initial condition. Inserting Eq. 77 into 112, we have Eq. 111 with $\xi=\left(6k^4-10k^3+3k^2+6k+2\right)/\left(12k^3\right)$ for local transitions. Such a transition pattern is a case of Ω. Inserting Eq. 77 into 114, we have Eq. 111 with $\xi=(k-1)/2$ for global transitions. Eq. 111 corresponds to Eq. 1 in the main text. For pairwise-comparison updating, analogously, we have

$$\rho_C>\rho_D \iff \xi\frac{\Delta b}{c}>1, \qquad [115]$$

where $\xi=\left(10k^2-4k+1\right)/\left(24k^2-12k\right)$ for local game transitions and $\xi=1/2$ for global game transitions. Eq. 115 corresponds to Eq. 2 in the main text.

With the same game transition pattern, in the competition between two stochastic strategies, i.e. s_p and s_q ($p>q$), from Eqs. 100 and 102, we have

$$\rho_{s_p}>\rho_{s_q} \iff \frac{b_1}{c}>k-\frac{(k+1)\left(p^2+q^2\right)+2pq-2}{2}\frac{\Delta b}{c} \qquad [116]$$

under death-birth updating and

$$\rho_{s_p}>\rho_{s_q} \iff \frac{p^2+q^2}{2}\frac{\Delta b}{c}>1 \qquad [117]$$

under birth-death or pairwise-comparison updating. Eq. 116 shows that for a large value of p, game transitions lower the threshold for $\rho_{s_p}>\rho_{s_q}$ relative to playing a fixed game. Eq. 117 shows that game transitions make it possible for cooperative stochastic strategies to be favored over less cooperative stochastic strategies under birth-death or pairwise-comparison updating, which has not been observed when players play a fixed game.

We next consider a game transition pattern under which the evolutionary outcome relies on the initial condition. The game transition matrices are shown in Eq. 74. $\mathbf{M}$ in Eq. 73 is given by

$$\mathbf{M} = \begin{array}{c} \\ E_{AA}^{(1)} \\ E_{AA}^{(2)} \\ E_{AB}^{(1)} \\ E_{AB}^{(2)} \\ E_{BB}^{(1)} \\ E_{BB}^{(2)} \end{array} \begin{array}{c} \begin{array}{cccccc} E_{AA}^{(1)} & E_{AA}^{(2)} & E_{AB}^{(1)} & E_{AB}^{(2)} & E_{BB}^{(1)} & E_{BB}^{(2)} \end{array} \\ \left(\begin{array}{cccccc} 1/2 & 0 & 1/2 & 0 & 0 & 0 \\ 0 & 1/2 & 0 & 1/2 & 0 & 0 \\ 1/3 & 0 & 1/3 & 0 & 1/3 & 0 \\ 0 & 1/3 & 0 & 1/3 & 0 & 1/3 \\ 0 & 0 & 1/2 & 0 & 1/2 & 0 \\ 0 & 0 & 0 & 1/2 & 0 & 1/2 \end{array} \right) \end{array}. \qquad [118]$$

Switching a few row entries and column entries, we have

$$\tilde{\mathbf{M}} = \begin{array}{c} \\ E_{AA}^{(1)} \\ E_{AB}^{(1)} \\ E_{BB}^{(1)} \\ E_{AA}^{(2)} \\ E_{AB}^{(2)} \\ E_{BB}^{(2)} \end{array} \begin{array}{c} \begin{array}{cccccc} E_{AA}^{(1)} & E_{AB}^{(1)} & E_{BB}^{(1)} & E_{AA}^{(2)} & E_{AB}^{(2)} & E_{BB}^{(2)} \end{array} \\ \left(\begin{array}{cccccc} 1/2 & 1/2 & 0 & 0 & 0 & 0 \\ 1/3 & 1/3 & 1/3 & 0 & 0 & 0 \\ 0 & 1/2 & 1/2 & 0 & 0 & 0 \\ 0 & 0 & 0 & 1/2 & 1/2 & 0 \\ 0 & 0 & 0 & 1/3 & 1/3 & 1/3 \\ 0 & 0 & 0 & 0 & 1/2 & 1/2 \end{array} \right) \end{array}. \qquad [119]$$

By analyzing $\tilde{\mathbf{M}}$, we get $E_{AA}^{(1)}, E_{AB}^{(1)}, E_{BB}^{(1)}$ belonging to one closed communicating class, and $E_{AA}^{(2)}, E_{AB}^{(2)}, E_{BB}^{(2)}$ belonging to the other class. Thus, the original system can be reduced into two subsystems, one consisting of $E_{AA}^{(1)}, E_{AB}^{(1)}, E_{BB}^{(1)}$ and the other consisting of $E_{AA}^{(2)}, E_{AB}^{(2)}, E_{BB}^{(2)}$. Here, we deal with the subsystem consisting of $E_{AA}^{(1)}, E_{AB}^{(1)}, E_{BB}^{(1)}$. Substituting $\mathbf{P}^{(2)}$, $\mathbf{P}^{(1)}$, and $\mathbf{P}^{(0)}$ into Eq. 42, we have $\dot{\mathbf{v}} = \bar{\mathbf{A}}\mathbf{v}$. $\bar{\mathbf{A}}$'s eigenvalues are $\lambda_1 = 0, \lambda_2 = -(k-2)/(kN), \lambda_3 = -(2k-2)/(kN)$, where λ_2 and λ_3 are negative for $k > 2$. Decomposing $\bar{\mathbf{A}}$ into $\bar{\mathbf{A}} = \mathbf{V}\mathbf{D}\mathbf{V}^{-1}$, where

$$\mathbf{V} = \begin{bmatrix} -\frac{p_A(kp_A-2p_A+1)}{(p_A-1)(k+2p_A-kp_A-1)} & -\frac{kp_A-2p_A+1}{k+2p_A-kp_A-1} & 1 \\ \frac{p_A(k-2)}{k+2p_A-kp_A-1} & -\frac{k+4p_A-2kp_A-2}{2(k+2p_A-kp_A-1)} & -1 \\ 1 & 1 & 1 \end{bmatrix}, \quad \mathbf{D} = \begin{bmatrix} 0 & 0 & 0 \\ 0 & -\frac{k-2}{kN} & 0 \\ 0 & 0 & -\frac{2k-2}{kN} \end{bmatrix}, \qquad [120]$$

we can get $p_{AA}^{(1)}(t)$, $p_{AB}^{(1)}(t)$ and $p_{BB}^{(1)}(t)$ as a function of time t, given by

$$\begin{bmatrix} p_{AA}^{(1)}(t) \\ p_{AB}^{(1)}(t) \\ p_{BB}^{(1)}(t) \end{bmatrix} = \mathbf{V}e^{\mathbf{D}t}\mathbf{V}^{-1} \begin{bmatrix} p_{AA}^{(1)}(t_0) \\ p_{AB}^{(1)}(t_0) \\ p_{BB}^{(1)}(t_0) \end{bmatrix}, \qquad [121]$$

where $p_{AA}^{(1)}(t_0), p_{AB}^{(1)}(t_0), p_{BB}^{(1)}(t_0)$ are the initial values of $p_{AA}^{(1)}, p_{AB}^{(1)}, p_{BB}^{(1)}$. When t approaches infinity, we have

$$p_{AA}^{(1)}(\infty) = \frac{(k-2)p_A^2 + p_A}{k-1} \left(p_{AA}^{(1)}(t_0) + 2p_{AB}^{(1)}(t_0) + p_{BB}^{(1)}(t_0) \right); \qquad [122a]$$

$$p_{AB}^{(1)}(\infty) = -\frac{(k-2)p_A^2 - (k-2)p_A}{k-1} \left(p_{AA}^{(1)}(t_0) + 2p_{AB}^{(1)}(t_0) + p_{BB}^{(1)}(t_0) \right); \qquad [122b]$$

$$p_{BB}^{(1)}(\infty) = \frac{(k-2)p_A^2 + (3-2k)p_A + k - 1}{k-1} \left(p_{AA}^{(1)}(t_0) + 2p_{AB}^{(1)}(t_0) + p_{BB}^{(1)}(t_0) \right). \qquad [122c]$$

Note that $p_{AA}^{(1)}(t_0) + 2p_{AB}^{(1)}(t_0) + p_{BB}^{(1)}(t_0)$ is the initial frequency of game 1, denoted by $p^{(1)}$. Then, we have $p_{AA}^{(1)}$, $p_{AB}^{(1)}$, and $p_{BB}^{(1)}$ as functions of p_A and $p^{(1)}$. Analogously, $p_{AA}^{(2)}$, $p_{AB}^{(2)}$, and $p_{BB}^{(2)}$ are functions of p_A and $p^{(2)}$. Substituting $p_{AA}^{(1)}$, $p_{AB}^{(1)}$, $p_{BB}^{(1)}$ and $p_{AA}^{(2)}$, $p_{AB}^{(2)}$, $p_{BB}^{(2)}$ into Eqs. 44a-44d, we can reduce Eq. 53 to

$$(k+1)\sum_{i=1}^{2} p^{(i)}R_i + (k-1)\sum_{i=1}^{2} p^{(i)}S_i - (k-1)\sum_{i=1}^{2} p^{(i)}T_i - (k+1)\sum_{i=1}^{2} p^{(i)}P_i > 0. \qquad [123]$$

For donation games, we have

$$\frac{b_1}{c} > k + p^{(2)} \frac{\Delta b_{12}}{c}. \qquad [124]$$

$\xi = -p^{(2)}$ shows that the evolutionary outcome is sensitive to the initial fractions of various games.

Based on Eqs. 86 and 87, we can perform the analogous study under global game transitions. With the game transition pattern given in Eq. 74, we have $\xi = -p^{(2)}$.

D. Evolutionary dynamics with probabilistic game transitions among three states ($n = 3$). All examples that we examine in the main text exhibit a deterministic game transition. That is, the game to be played in the next time step is not probabilistic. In this section, we present an example with stochastic game transitions among three states. Game 1 is the most valuable and game 3 is the least valuable, i.e., $b_1 > b_2 > b_3$. The game transition matrices are given by

$$\mathbf{P}^{(2)} = \begin{bmatrix} 1 & 0 & 0 \\ p & 1-p & 0 \\ p & 0 & 1-p \end{bmatrix}, \quad \mathbf{P}^{(1)} = \begin{bmatrix} 1-p & p & 0 \\ 0 & 1 & 0 \\ 0 & p & 1-p \end{bmatrix}, \quad \mathbf{P}^{(0)} = \begin{bmatrix} 1-p & 0 & p \\ 0 & 1-p & p \\ 0 & 0 & 1 \end{bmatrix}. \qquad [125]$$

Mutual cooperation (mutual defection) is prone to yield game 1 (game 3) and unilateral cooperation/defection a moderately-valuable game 2. The game transition occurs with a probability p and players play the old game with a probability $1-p$. For $p = 0$, by virtue of the approach in Section 2, we know that the evolutionary outcome relies on the initial condition. We can refer to the example in Section 4C to derive the evolutionary outcome. For $p > 0$, the evolutionary outcome is independent of the initial condition. Note that $p = 1$ corresponds to the deterministic case. Under death-birth updating, the general rule for cooperation to be favored over defection is

$$\rho_C > \rho_D \iff \frac{b_1}{c} > k - \xi_2 \frac{\Delta b_{12}}{c} - \xi_3 \frac{\Delta b_{13}}{c}. \qquad [126]$$

For local game transitions, we have

$$\xi_2 = \frac{\left(6k^5 - 15k^4 + 24k^3 - 24k^2 + 15k - 6\right)p^2 + \left(4k^4 - 21k^3 + 32k^2 - 19k + 14\right)p - 2k^2 + 8k - 8}{\left(12k^4 - 18k^3 + 30k^2 - 18k + 12\right)p^2 + \left(18k^3 - 36k^2 + 36k - 24\right)p + 6k^2 - 18k + 12} \qquad [127]$$

and

$$\xi_3 = \frac{\left(k^4 + 2k^2 - 7k + 2\right)p - 2k^2 + 5k - 2}{\left(12k^4 - 18k^3 + 30k^2 - 18k + 12\right)p^2 + \left(18k^3 - 36k^2 + 36k - 24\right)p + 6k^2 - 18k + 12}. \qquad [128]$$

Here, we analyze how the probabilistic transition measured by p affects the critical benefit-to-cost ratio $(b_1/c)^*$. Analyzing $(b_1/c)^* = k - \xi_2 \Delta b_{12}/c - \xi_3 \Delta b_{13}/c$, we get a threshold

$$\left(\frac{\Delta b_{13}}{\Delta b_{12}}\right)^* = 5 - \frac{3\left(k+4\right)\left(k^2 + 2k - 2\right)}{2k^5 - 3k^4 + 6k^2 + 2k - 8}. \qquad [129]$$

If $\Delta b_{12}/\Delta b_{13}$ is lower than $(\Delta b_{12}/\Delta b_{13})^*$, $(b_1/c)^*$ decreases monotonically in p. If $\Delta b_{12}/\Delta b_{13}$ is larger than $(\Delta b_{12}/\Delta b_{13})^*$, $(b_1/c)^*$ is a non-monotonic function of p. Specifically, as p increases starting from a value slightly larger than 0, $(b_1/c)^*$ decreases first and then increases. The optimal transition probability p for collective cooperation is

$$p^* = \frac{\phi_2 r + \phi_3 + \sqrt{\phi_4 r^2 + \phi_5 r + \phi_6}}{\phi_0 r + \phi_1}, \qquad [130]$$

where

$$\begin{aligned} \phi_0 &= k^4 + 2k^2 - 7k + 2; \\ \phi_1 &= -5k^4 + 6k^3 - 4k^2 + 11k + 2; \\ \phi_2 &= \left(2k-1\right)\left(k-2\right); \\ \phi_3 &= \left(k-2\right)\left(3k^2 - 4k - 1\right); \\ \phi_4 &= \left(-k^3 - 4k^2 + 2k + 2\right)\phi_7; \\ \phi_5 &= \left(k^3 + 13k^2 - 8k - 8\right)\phi_7; \\ \phi_6 &= \left(2k^3 - 10k^2 + 8k + 8\right)\phi_7; \\ \phi_7 &= \frac{-k^2\left(k-2\right)\left(k^2 - k - 1\right)\left(k^2 + 2k - 2\right)}{\left(2k^2 - k + 2\right)\left(k^2 - k + 1\right)}. \end{aligned} \qquad [131]$$

Depending on the variations in different games, probabilistic game transitions can strengthen the promotive effects of game transitions on the evolution of cooperation in a few cases, whereas weaken them in other cases. The conclusion holds under other updating rules like imitation and pairwise-comparison updating.

For global game transitions, the related parameters are $\xi_2 = (k-1)/2$ and $\xi_3 = 0$. In this case, probabilistic transitions do not alter the effects of game transitions on the evolution of cooperation.

References

1. Nowak MA, Sasaki A, Taylor C, Fudenberg D (2004) Emergence of cooperation and evolutionary stability in finite populations. *Nature* 428(6983):646–650.
2. Ewens WJ (2004) *Mathematical Population Genetics. I. Theoretical Introduction.* (New York: Springer).
3. Wu B, Altrock PM, Wang L, Traulsen A (2010) Universality of weak selection. *Phys. Rev. E* 82(4):046106.
4. Wu B, García J, Hauert C, Traulsen A (2013) Extrapolating weak selection in evolutionary games. *PLoS Comput. Biol.* 9(12):e1003381.
5. Ohtsuki H, Hauert C, Lieberman E, Nowak MA (2006) A simple rule for the evolution of cooperation on graphs and social networks. *Nature* 441(7092):502–505.
6. Khalil HK (2001) *Nonlinear Systems.* (Prentice Hall).
7. Gardiner CW (2004) *Handbook of Stochastic Methods.* (Springer).
8. McAvoy A, Hauert C (2015) Asymmetric evolutionary games. *PLoS Comput. Biol.* 11(8):e1004349.
9. Su Q, Zhou L, Wang L (2019) Evolutionary multiplayer games on graphs with edge diversity. *PLoS Comput. Biol.* 15(4):e1006947.
10. Tarnita CE, Ohtsuki H, Antal T, Fu F, Nowak MA (2009) Strategy selection in structured populations. *J. Theor. Biol.* 259(3):570–581.
11. Erdös P, Rényi A (1960) On the evolution of random graphs. *Publ. Math. Inst. Hung. Acad. Sci.* 5:17–61.
12. Barabási AL, Albert R (1999) Emergence of scaling in random networks. *Science* 286(5439):509–512.
13. Albert R, Barabási AL (2002) Statistical mechanics of complex networks. *Rev. Mod. Phys.* 74(1):47–97.

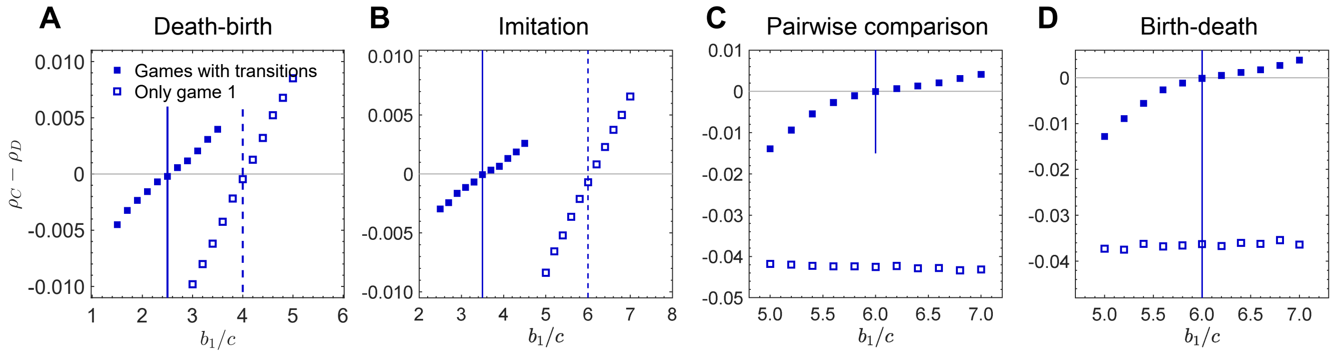

Fig. S1. Game transitions can promote cooperation. We study the transition between two donation games: a cooperator pays a cost c to bring its opponent a benefit b_1 in game 1 or b_2 in game 2; defectors forgo this donation. b_1 is larger than b_2. Mutual cooperation leads to game 1 and other action profiles lead to game 2. We examine death-birth (**A**), imitation (**B**), pairwise-comparison (**C**), and birth-death (**D**) updating on random regular graphs. The cross points of the dots and the horizontal lines mark the critical benefit-to-cost ratios for cooperation to be favored over defection, i.e. $\rho_C > \rho_D$, by numerical simulations. The vertical lines give the analytical critical benefit-to-cost ratios. Under death-birth and imitation updating, game transitions reduce the critical benefit-to-cost for $\rho_C > \rho_D$. Under pairwise-comparison and birth-death updating, game transitions make it possible for $\rho_C > \rho_D$. We take $N = 500$, $k = 4$, $\delta = 0.01$, $c = 1$. Other parameters: $b_2 = b_1 - 1$ for death-birth and imitation updating, $b_2 = 4$ for pairwise-comparison and birth-death updating. Each simulation runs until the population reaches fixation and each point is averaged over 10^6 runs.

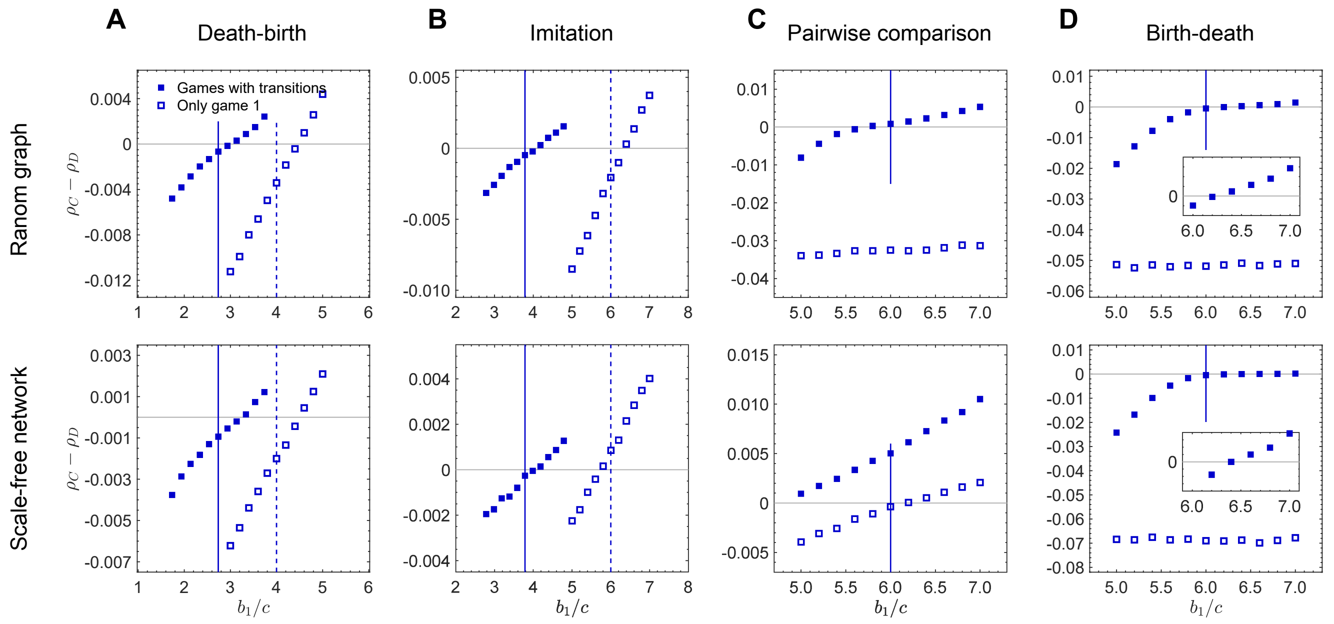

Fig. S2. Game transitions can promote cooperation on social networks. We study the transition between two donation games: a cooperator pays a cost c to bring its opponent a benefit b_1 in game 1 or b_2 in game 2; defectors pay no costs and provide no benefits. b_1 is larger than b_2. Mutual cooperation allows for game 1 and other action profiles lead to game 2. We examine death-birth (**A**), imitation (**B**), pairwise-comparison (**C**), and birth-death (**D**) updating on random graphs (11) and scale-free networks (12, 13). The cross points of the dots and the horizontal lines mark the critical benefit-to-cost ratios for cooperation to be favored over defection by numerical simulations. The vertical lines give the analytical critical benefit-to-cost ratios based on random regular graphs. The average degree of the random regular graph and the scale-free networks is 4. Other parameters are the same as those in Fig. S1. Game transitions reduce the critical benefit-to-cost for the success of cooperators ($\rho_C > \rho_D$).

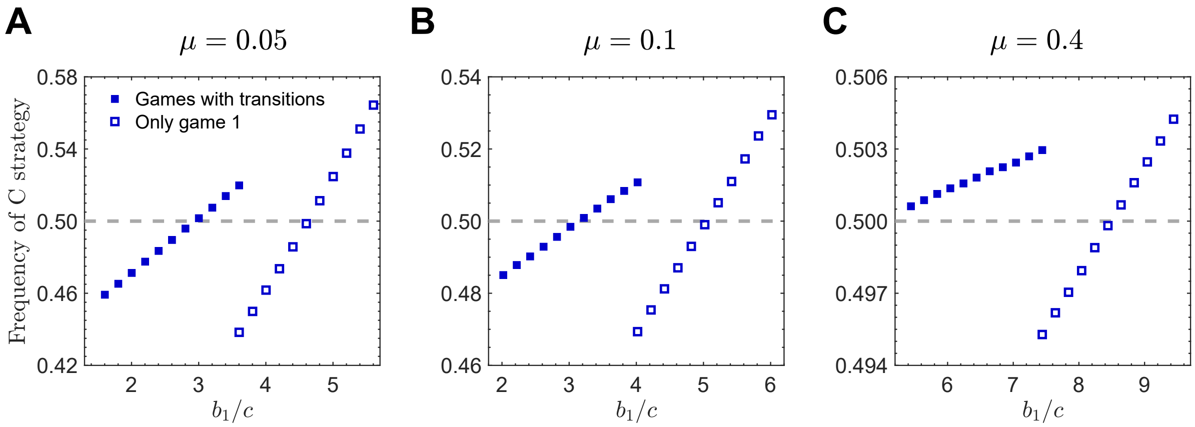

Fig. S3. Game transitions can promote cooperation in the presence of mutation or random strategy exploration. We study the transition between two donation games: a cooperator pays a cost c to bring its opponent a benefit b_1 in game 1 or b_2 in game 2; defectors pay no costs and provide no benefits. b_1 is larger than b_2. Mutual cooperation allows for game 1 and other action profiles lead to game 2. We investigate death-birth updating on random regular graphs. With probability $1-\mu$, the empty site is occupied by the neighbor's offspring. With probability μ, the empty is occupied by a cooperator or a defector with equal probability. Here, the frequency of cooperative strategies $\langle f_C \rangle$ is used to measure the success of cooperators. Cooperation is favored over defection if $\langle f_C \rangle > 1/2$. We obtain each data point by averaging $\langle f_C \rangle$ in 100 independent runs. For each run, $\langle f_C \rangle$ is obtained by averaging the frequency of cooperative strategies in the last 2×10^7 time steps. We take $N = 500$, $k = 4$, $b_2 = b_1 - 1$, $\delta = 0.01$, and $c = 1$. Other parameters: $\mu = 0.05$ (**A**), $\mu = 0.1$ (**B**), and $\mu = 0.4$ (**C**). The cross points of the dots and the horizontal lines mark the critical benefit-to-cost ratios for cooperation being favored over defection ($\langle f_C \rangle > 1/2$). Game transitions reduce the critical benefit-to-cost for the success of cooperators.

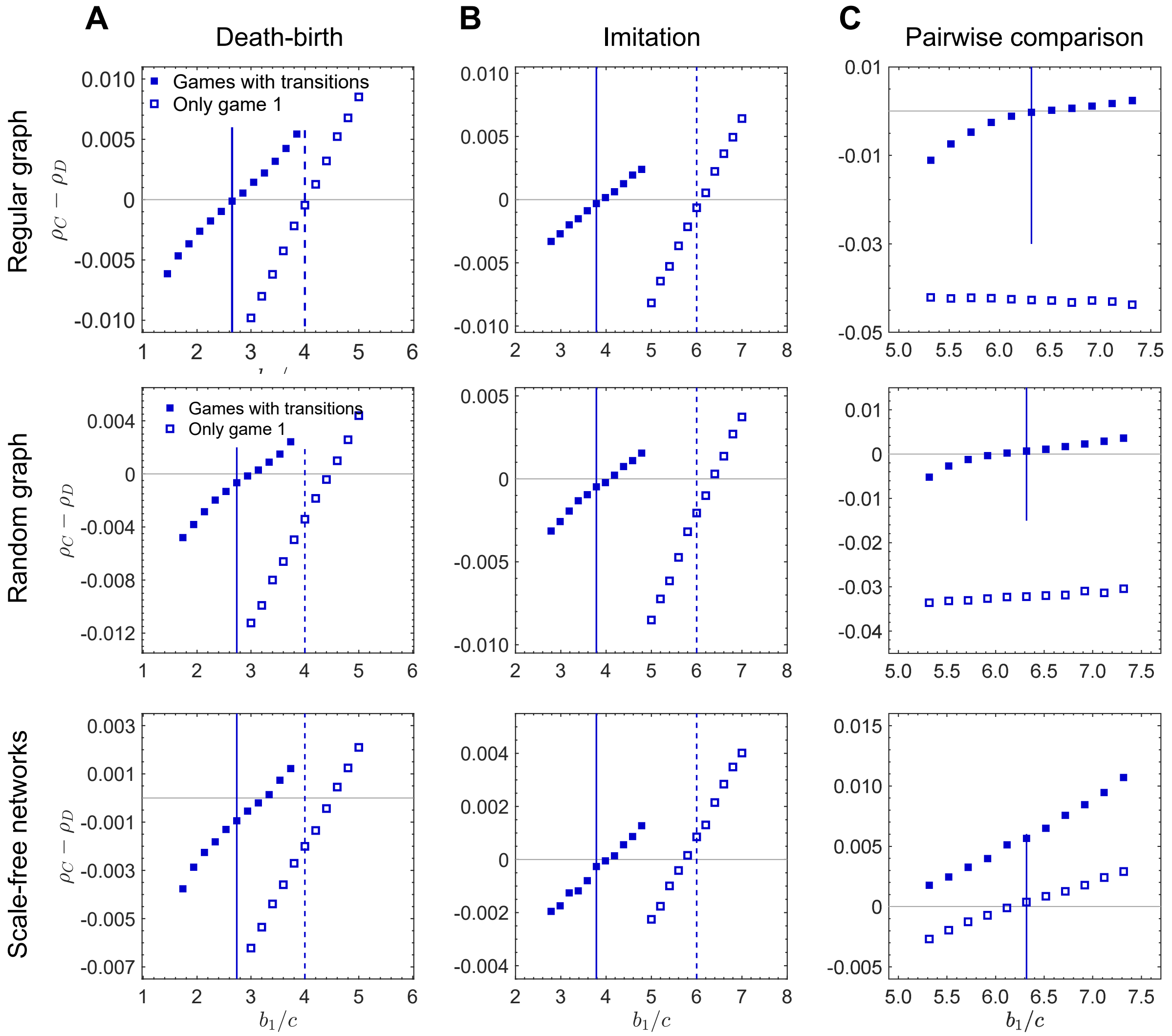

Fig. S4. Local game transitions can promote cooperation. We study the transition between two donation games: a cooperator pays a cost c to bring its opponent a benefit b_1 in game 1 or b_2 in game 2; defectors pay no costs and provide no benefits. b_1 is larger than b_2. Mutual cooperation allows for game 1 and other action profiles lead to game 2. We examine death-birth (**A**), imitation (**B**), and pairwise-comparison (**C**) on random regular graphs, random graphs, and scale-free networks. The cross points of the dots and the horizontal lines mark the critical benefit-to-cost ratios for cooperation to be favored over defection by numerical simulations. The vertical lines give the analytical critical benefit-to-cost ratios. Game transitions reduce the critical benefit-to-cost for the success of cooperators ($\rho_C > \rho_D$). All parameters are the same as those in Fig. S1.

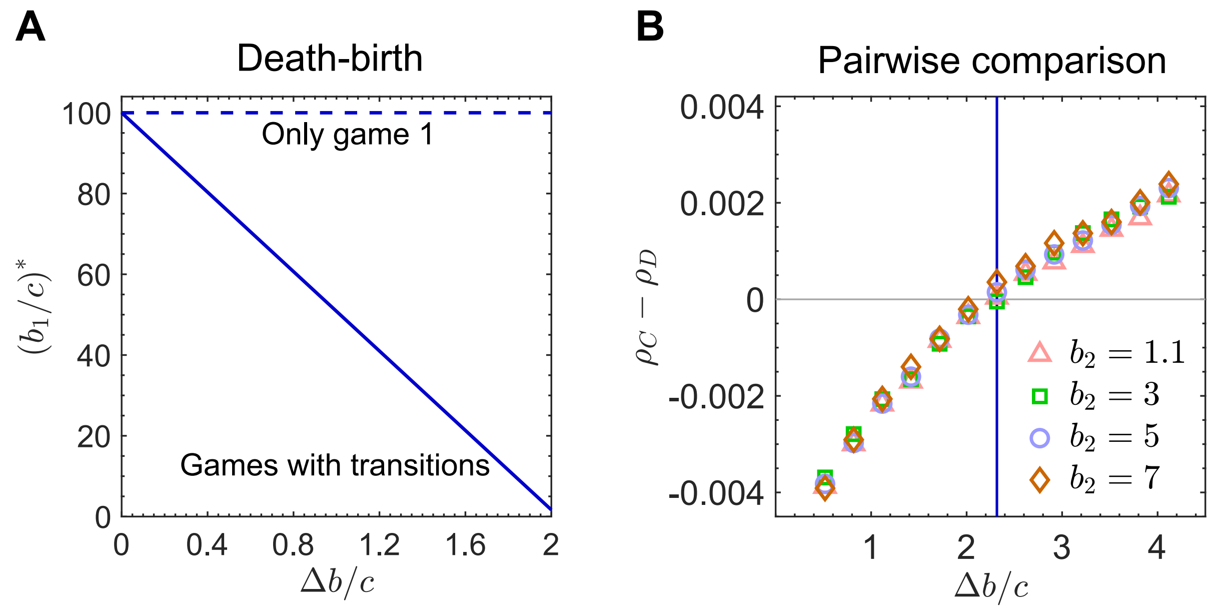

Fig. S5. With local game transitions a small variation in different games can promote cooperation markedly. We study the transition between two donation games: a cooperator pays a cost c to bring its opponent a benefit b_1 in game 1 or b_2 in game 2; defectors forgo the helping behavior. b_1 is larger than b_2. Mutual cooperation allows for game 1 and other action profiles lead to game 2. We examine death-birth (**A**) and pairwise-comparison (**B**) updating on random regular graphs. Under death-birth updating, a small difference between b_1 and b_2 ($\Delta b = b_1 - b_2$) greatly reduces the critical benefit-to-cost ratio (**A**). Under pairwise-comparison updating, the difference between games, $b_1 - b_2$, rather than the individual value of b_1 and b_2, determines the success of cooperators (**B**). Apart from b_1 and b_2, all other parameters follow Fig. S1.
